# Mechanistic insights and process optimization of pristine corn husk biosorbent for sustainable and cost effective removal of cationic dyes from waste water

**DOI:** 10.1038/s41598-026-45206-9

**Published:** 2026-04-13

**Authors:** Magda A. Akl, Aya G. Mostafa, Asmaa A. Serage, Noha A. Abd-Rabo

**Affiliations:** Department of Chemistry, Faculty of Science, Mansoura University, Mansoura, 35516 Egypt

**Keywords:** Corn husk, Cationic dyes, Basic fuchsin (BF), Crystal violet (CV), Adsorption, RSM, Wastewater treatment, Chemistry, Environmental sciences, Materials science

## Abstract

Ensuring safe and accessible drinking water requires effective wastewater treatment. In this work, pristine corn husk biosorbent (CH) was employed as a low-cost material for the removal of Basic Fuchsin (BF) and Crystal Violet (CV) dyes. The CH biosorbent was comprehensively characterized through elemental analysis, scanning electron microscopy (SEM), N₂ adsorption–desorption isotherms, Brunauer–Emmett–Teller (BET) surface area measurements, Fourier transform infrared spectroscopy (FTIR), point of zero charge (pH_PZC_), and thermogravimetric analysis (TGA). Batch adsorption studies were carried out to evaluate the influence of various operating parameters, such as CH biosorbent dosage, contact time, temperature, and initial dye concentration. The adsorption kinetics were found to fit the pseudo-second-order model, while the equilibrium isotherm data aligned best with the Langmuir model, showing high correlation coefficients (R² ≥ 0.999) and low error values compared to other tested models. The maximum adsorption capacities were determined to be 77.3 mg/g for BF and 88.8 mg/g for CV. Thermodynamic analysis indicated that the uptake of both dyes onto the CH biosorbent is spontaneous and endothermic. Response surface methodology (RSM) with a central composite design (CCD) was employed to fine-tune the reaction parameters and optimize the adsorption process. The adsorption mechanism is attributed to a combination of electrostatic attraction, π–π stacking, n–π interactions, hydrogen bonding, and pore diffusion. The CH biosorbent demonstrated good reusability, maintaining its performance through five regeneration cycles. Additionally, antibacterial activity tests of the CH biosorbent were conducted before and after dye uptake to assess potential toxicity. The CH biosorbent successfully removed more than 90% of CV and BF from real water samples, highlighting its promise as a sustainable and environmentally friendly biosorbent for water purification.

**Supplementary Information:**

The online version contains supplementary material available at 10.1038/s41598-026-45206-9.

## Introduction

Water is a vital component of the universe and plays a crucial role in maintaining the proper functioning of Earth’s ecosystems. Despite its importance, many regions around the world still lack access to safe drinking water. This shortage leads to widespread health problems, as millions of people suffer from illnesses caused by unsafe water and poor sanitation^1^. Waterborne diseases, including intestinal parasitic infections, bacterial diseases, and enteric viruses, are common in such conditions.

Furthermore, water quality is deteriorating at an alarming rate due to contamination^2^. The main causes include rapid population growth, industrialization, urbanization, domestic and agricultural activities, as well as other geological, environmental, and global changes. Water pollution has thus become a serious challenge, directly threatening human health, and is expected to worsen in the coming decades^3^.

More than 700 organic and inorganic pollutants have been found in water, as well as microbial populations. Because they are extremely toxic and carcinogenic, some of these pollutants, both organic and inorganic, are harmful^4^. The increasing discharge of dye-containing wastewater from various industrial sectors every year has become a major environmental concern worldwide. Dyes are synthetic organic compounds widely used in the textile, leather, food, cosmetic, and paper industries.These dye-contaminated effluents are not only aesthetically unpleasant but also pose severe environmental and health hazards due to their toxicity, potential carcinogenicity, and resistance to biodegradation^5,6^.

Cationic dyes, also known as basic dyes, are synthetic organic compounds that carry a positive charge in aqueous solutions. Cationic dyes are widely used in the textile, paper, plastic, cosmetic, and biological industries due to their brilliant colors, high tinctorial strength, and fastness on synthetic fibres. Crystal violet and Basic Fuchsin are two of the most widely studied cationic dyes. Crystal violet is a triphenylmethane dye. Also widely used for textile dyeing, microbiological staining, and as a model pollutant in environmental studies due to its chemical stability and intense violet color^7,8^. Similarly, BF is a magenta colored dye from the triphenylmethane family. Also used for biological staining and as a laboratory reagent. CV and BF are both toxic, non-biodegradable, and potentially carcinogenic, so removing them from wastewater is an environmental priority^9^.

These challenges have driven research efforts toward advanced techniques for the efficient and eco-friendly elimination of such persistent dyes from industrial effluents. Common methods used for wastewater treatment include coagulation, flocculation, sedimentation, chemical oxidation, membrane filtration, biological treatment, adsorption, and advanced oxidation processes^10,11^. These methods are selected based on the type and concentration of pollutants present, and play a vital role in reducing the environmental impact of contaminated water. While each method has its advantages, it may also have limitations, such as high operational costs, excessive energy consumption, or inadequate pollutant removal.

In contrast, adsorption has emerged as one of the most promising and extensively researched wastewater treatment techniques. It involves the accumulation of pollutants on the surface of solid materials (adsorbents). It is well-known for its simplicity of use, low cost, and ability to remove a wide range of contaminants at low concentrations. Due to its high efficiency, adaptability, and adsorption capabilities, it is increasingly regarded as a sustainable and practical solution for treating wastewater^12^.

Biosorbents play an important role in wastewater treatment and have received increased attention due to their economic and environmental advantages. Agricultural residues such as corn husk, rice husks, wheat bran, fruit peels, and sugarcane bagasse, as well as industrial biosorbents, are rich in carbon and functional groups, making them ideal for adsorption processes. These biosorbents are often available in large quantities for little or no cost, making them very attractive alternatives to conventional adsorbents such as activated carbon, which can be expensive^13^. Their porous structure and surface chemistry enable them to effectively adsorb a wide range of pollutants, including dyes, heavy metals, and organic compounds. Incorporating biosorbents into adsorption processes helps promote sustainable waste management by converting potential environmental liabilities into valuable resources^14^. This approach is consistent with the principles of green chemistry and the circular economy, in which waste materials are reused or repurposed rather than discarded. In addition to pollutant removal using biosorbents in adsorption, the overall environmental footprint of the treatment process is reduced. As global industries strive for more sustainable, eco-efficient practices, utilizing agricultural, industrial biosorbents in adsorption processes represents a promising strategy for cleaner water, reduced solid waste, benefiting both the environment and the economy^15^.

Among recent adsorption studies, Akl et al. introduced pristine corn kernels (CK) as a pH-responsive biosorbent for removing Acid Green 20 (AG20) and Crystal Violet (CV) dyes. The material achieved adsorption capacities of 85.74 mg/g for AG20 and 61.1 mg/g for CV, with over 90% removal from real water samples. Adsorption followed the pseudo-second-order kinetic and Langmuir isotherm models, indicating spontaneous and endothermic behavior. The CK biosorbent was reusable up to five cycles, confirming its promise as a sustainable and eco-friendly material for wastewater treatment^16^.

In recent years, considerable attention has been directed toward developing sustainable and bio-based adsorbents derived from agricultural residues and natural polymers for the removal of hazardous dyes from wastewater. Chitosan-based adsorbents have demonstrated effective dye uptake^17^, while sodium alginate biopolymer matrices have shown versatility toward a broad range of dyes^18^. Various agro-waste materials, including Azadirachta indica sawdust^19^, modified watermelon rind^20^, alkalinated pumpkin seed shells^21^, activated rice husk^22^, almond shell biosorbents^23^, and banana peel-derived materials^24^, have also exhibited promising adsorption performance at relatively low cost. Additionally, activated carbon prepared from agricultural and industrial wastes^25^ and cationized corn husk (CCH)^26^ have been investigated to enhance dye removal efficiency. However, most studies on corn husk have focused on chemically or structurally modified forms, which may require additional processing steps and cost. In this context, the present study examines pristine corn husk (CH) as a low-cost biosorbent for the removal of BF and CV, aiming to evaluate its performance without chemical modification.

In response, research has increasingly focused on eco-friendly and cost-effective alternatives, particularly those derived from agricultural biosorbents. One such material is corn husk (CH), which is primarily composed of cellulose (35–45%), hemicellulose (25–35%), as shown in Fig.S1, and lignin (15–20%), making it a typical lignocellulosic material. Cellulose provides structural strength, whereas hemicellulose increases flexibility and water affinity. Lignin facilitates rigidity and hydrophobicity. Minor components include ash (1–5%), extractives (1–3%), such as waxes and resins^27^. This composition makes CH suitable for biosorption and other environmental applications.

In the present study, we propose a novel, sustainable, and cost-effective strategy by employing CH as a biosorbent, investigating its efficiency in removing more than one cationic dye, specifically CV and BF, from aqueous solutions without any physical or chemical modification. What makes this work distinctive is its demonstration of the high adsorption performance of untreated raw CH, highlighting its potential as a green and efficient biosorbent for dye-contaminated wastewater treatment.

The key advantages of this approach lie in its simplicity, affordability, and environmental compatibility, as it relies on readily available agrowaste without requiring any chemical processing or additional equipment. Moreover, the ability of the CH biosorbent to simultaneously remove more than one dye enhances its practical applicability in real-world industrial wastewater treatment.

This study also addresses a minor research gap, as most previous studies have focused on modified or treated biosorbents to enhance adsorption efficiency, while raw, untreated materials have received little attention despite their simplicity and low cost. Therefore, this work provides a new perspective on the potential of pristine CH as a natural, sustainable, and efficient biosorbent for the treatment of dye-contaminated wastewater. Although the raw biosorbent has shown promising performance, certain aspects may benefit from further Optimization in future studies. This feature represents a distinct advantage of the proposed approach, as it combines efficiency, simplicity, and sustainability without requiring complex chemical modifications. These characteristics also provide clear environmental and economic benefits, making it a promising and practical option for treating dye-contaminated wastewater.

The present study aims to achieve several objectives related to pristine CH biosorbent: **i-**Preparation, and characterization of pristine CH biosorbent (CH) utilizing FTIR, BET, pH_PZC_, elemental analysis, TGA and SEM; **ii-**Experimentation with different cationic dyes CV, and BF adsorption using CH in aqueous solutions; **iii-** Analyzing the various experimental factors, such as adsorbent dose, pH, initial dye concentrations, and ionic strength; **iv-**Performing kinetic, and adsorption isotherm studies to comprehend the adsorption mechanism, and the maximum adsorption capacity of CH; **v**-Statistical analysis of the isotherm, and kinetic models using the chi-square statistic (χ2), mean square error (MSE), hybrid error, and the sum of squares error (SSE) error functions; **vi**-Application of the CH biosorbent for the removal of CV, and BF dyes from real water samples; **vii-** Response surface methodology (RSM) with Box Behnken Design (BBD) to fine-tune the reaction parameters in order to optimize the adsorption process; **viii-**Elucidation of the adsorption mechanism of CV, and BF onto CH; **ix-** Adsorption of dye in the mixture of CV, and BF using CH biosorbent.

## Experimental

### Materials and chemicals

Corn husk (CH) wascollected from a local market in Mansoura, Egypt. All chemicals and reagents used in this study were of analytical grade and purchased from Sigma-Aldrich, Egypt.

CV (C₂₅H₃₀ClN₃, purity 90%, CAS No. 548-62-9, Sigma-Aldrich, Egypt) and BF(C₂₀H₂₀ClN₃, purity 88%, CAS No. 632-99-5, Sigma-Aldrich, Egypt) were used as model cationic dyes (Table 1). Stock solutions of CV and BF (1000 mg/L) were prepared separately by dissolving 1 g of each dye in 1 L of distilled water. Sodium chloride (NaCl, ≥ 99.0%, CAS No. 7647-14-5, Sigma-Aldrich, Egypt), sodium hydroxide (NaOH, ≥ 99.5%, CAS No. 1310-73-2, Sigma-Aldrich, Egypt), sodium carbonate (Na₂CO₃, ≥ 99.5%, CAS No. 497-19-8, Sigma-Aldrich, Egypt), sodium acetate (CH₃COONa, ≥ 99.0%, CAS No. 127-09-3, Sigma-Aldrich, Egypt), sodium bicarbonate (NaHCO₃, ≥ 99.7%, CAS No. 144-55-8, Sigma-Aldrich, Egypt), sodium nitrate (NaNO₃, ≥ 99.0%, CAS No. 7631-99-4, Sigma-Aldrich, Egypt), potassium chloride (KCl, ≥ 99.0%, CAS No. 7447-40-7, Sigma-Aldrich, Egypt), and ethanol (99.0%, CAS No. 64-17-5, Sigma-Aldrich, Egypt) were used as received.

**Table 1:** Structure of dyes.

Dyes	Chemical formula	Chemical structure	Molecular weight	Molecular diameter
**CV**	(C_25_N_3_H_30_Cl)		407.98 g/mol	14 Å.
**BF**	(C_20_H_20_ClN_3_)		337.85 g/mol	13 Å−18Å.

### Preparation of the pristine corn husk (CH) biosorbent

The collected CH was ground in a mill and sieved to a mesh size of 0.8–1 mm after being cleaned of all impurities using ethanol and distilled water. They were then dried in an oven at 60 °C until a stable weight was reached.

### Characterization and instrumentation

The characterization of the CH biosorbent involved several analytical techniques. The Brunauer–Emmett–Teller (BET) method was applied to calculate the particular surface area of the CH biosorbent using [Quanta Chrome Touch Win™]. The elemental composition (C, N, H) of CH biosorbent, (CH-CV), and (CH- BF) was analyzed using a Costech ECS-4010 elemental analyzer. The surface morphology of the CH biosorbent was examined by scanning electron microscopy (SEM, model JSM-T 220 A, JEOL, Japan). Fourier transform infrared spectroscopy (FTIR) analysis using a Nicolet FTIR spectrophotometer (Perkin-Elmer Co., USA) to investigate the functional groups present in the samples was obtained in the range of 400–4000 cm⁻¹. A thermogravimetric analyzer (TGA-50 Shimadzu) was used to record the thermograms of the CH biosorbent by heating an aluminum pan between 30 and 800 degrees Celsius at a rate of 15 degrees Celsius. The adsorption spectra of the cationic dye CV and BF, as well as their mixtures, were measured using a UV-Vis spectrophotometer (JENWAY Co., Ltd., UK) with quartz cells over a wavelength range of 300–800 nm. The maximum absorbance wavelengths (λ_max_) were identified as 590 nm for CV and 545 nm for BF.

In addition, Response Surface Methodology (RSM) with central composite design (CCD) was employed as a statistical and mathematical tool to model, analyze, and fine-tune the reaction parameters in order to optimize the adsorption process. RSM enabled the evaluation of the combined effects of factors such as pH and initial dye concentration, the identification of optimal adsorption conditions, and the visualization of variable interactions through response surface plots, while significantly reducing the number of experimental runs required.

To determine the point of zero charge (pH_pzc_) for the CH biosorbent, 0.01 g of CH was added to 25 mL of 0.01 M NaCl solution, with pH adjusted between 2 and 12 using 0.1 M HCl or 0.1 M NaOH. Each flask received 0.1 g of CH, and the suspensions were allowed to stand for 48 h. The final pH (pH_f_) was measured, and the difference ΔpH = pH_f_ – pH_i_ was calculated. The pH_pzc_ was then determined from the intersection point (ΔpH = 0) on the plot of ΔpH versus initial pH (pH_i_), following the standard method^28^.

### Optimization of the adsorption process using response surface methodology (RSM)

The adsorption experiments of cationic dyes (CV and BF) were performed in 125 mL stoppered and transparent bottles containing 10 mL of cationic dye solution, and a dosage of CH. The maximum adsorption spectra of cationic dye (CV) is (590 nm), and BF is (545 nm). Batch sorption experiments were performed by shaking a known amount (0.01 g) of CH with 10 mL of BF solution (100 mg/L) and CV solution (100 mg/L) in 150 mL stopped bottles. The bottles were then shaken at 200 rpm on a thermostatic shaker set to room temperature. After reaching equilibrium, the solutions were centrifuged at 3,000 rpm. Then, the supernatant solution containing the remaining dye from each was measured at its respective λ_max_. Various parameters affecting the adsorption of CV and BF were investigated, like pH (from 2 to 10), temperature (25–50 °C), dose of CH (0.01–0.05 g), contact time(10–210 min), initial concentrations of (50–300 mg/L), and ionic strength. The pollutants removal percentage (R%) and adsorption capacity (q_e_, (mg/g)) were calculated using Eq. (1), and (2)^29^.12

where *C*_*o*_(mg/L) and *C*_*i*_ (mg/L) are the equilibrium dye concentrations after adsorption and the initial dye concentrations, respectively. wt is the mass of the CH adsorbent (g), and V (L) is the volume of the dye solution, q_e_ is the amount of dye adsorbed (mg/g).

Response Surface Methodology (RSM) is a commonly employed approach in experimental design for process analysis and modelling. RSM enables the examination of how various influencing factors interact with desired outcomes, requiring a minimal number of specified trials to determine optimal process conditions. Parameters such as pH, initial concentration were identified as the most significant factors. They were assessed at three different levels to evaluate the adsorption efficiency of the CH biosorbent, as detailed in Table 2. The Central Composite Design (CCD) was applied within the Response Surface Methodology (RSM) framework using Design Expert software (version 19). For an experimental design involving two independent factors, a total of 13 randomized runs were generated, consisting of four factorial points, four axial points, and five replicated center points to accurately estimate the experimental error and detect curvature in the response. Analysis of variance (ANOVA) was used to evaluate the significance of the linear, quadratic, and interaction effects of the studied variables. In this study, CCD was applied to investigate and optimize the Removal of CV and BF dyes using CH, and the significance of the process variables and the developed model was analyzed using ANOVA (analysis of variance).

**Table 2:** Experimental variables and their respective levels for the adsorption of dyes.

Independent variable	Coded	Unit	Factorial and center level		
			Low (−1)	Center (0)	High (+ 1)
pH	A	-	2	6	10
Initial concentration	B	mg/L	50	100	150

In this study, a central composite design (CCD) was employed to investigate the effect of two independent variables A and B for dyes as presented in Eq. (3) on the response Y. A second-order (quadratic) response surface model was used to describe the relationship between the factors and the response. The full quadratic model is expressed as:

3 $${\mathrm{Y}}\,=\,{\beta _0}\,+\,{\beta _1}{\mathrm{A}}\,+\,{\beta _2}{\mathrm{B}}\,+\,{\beta _{12}}{\mathrm{AB}}\,+\,{\beta _{11}}{{\mathrm{A}}_2}\,+\,{\beta _{22}}{{\mathrm{b}}_2}\,+\,\varepsilon$$

where Y represents the predicted response, β_0_ is the intercept, β_1_and β_2_ are the linear coefficients, β_12_ is the interaction coefficient β_11_ and β_22_ are the quadratic coefficients, and is the residual error. This model allows estimation of both linear and non-linear effects, as well as the interaction between the two factors, enabling optimization of the experimental conditions^30^.

### The isotherm and kinetic models

The isotherm studies for CV and BF adsorption were carried out by inserting 0.01 g of CH biosorbent in a succession of bottles containing CV and BF dye solutions. The initial concentration of the dye was between 50 and 300 mg/L. For 120 min, these bottles were shaken at 200 rpm at 25 °C for CV at pH 6, and BF at pH 8 in a thermostat shaker. The linear form of the Freundlich and Langmuir isothermal models was used, and the parameters were determined using Eqs. (4), (5), respectively^31,32^.

4 $$\:\frac{\mathrm{C}\mathrm{o}}{\mathrm{q}\mathrm{e}}=\frac{1}{{\mathrm{k}}_{\mathrm{L}}{\mathrm{q}}_{\mathrm{m}}}+\frac{\mathrm{C}\mathrm{o}}{{\mathrm{q}}_{\mathrm{m}}}$$

5 $$\:\mathrm{ln}{\mathrm{q}}_{\mathrm{e}}=\mathrm{ln}\mathrm{k}+\frac{1}{\mathrm{n}}\mathrm{I}\mathrm{n}{\mathrm{C}}_{\mathrm{e}\:}$$

As C_o_ (mg/L) is the concentration of BF and CV at equilibrium, q_e_ (mg/g) is the adsorbed dyes amount onto surface of CH biosorbent at equilibrium, q_m_ (mg/g) represents the system’s maximum adsorption uptake, 1/n is the heterogeneity factor, K_L_ (L/mg), and K_F_ (mg/g) (L/mg)^1/n^ are the Langmuir, and Freundlich constants, respectively. In addition, the characteristics of the Langmuir isotherm, R_L_, the separation factor, and a dimensionless constant are defined as:

6 $$\:{R}_{L}=\frac{1}{1+{k}_{L}{C}_{0}}$$

When R_L_ is between 0 and 1, the adsorption process is favourable. It is unfavourable when R_L_ exceeds one, irreversible when R_L_ is zero, and linear when R_L_ equals one. Analyzing the generated data reveals that the R_L_values are between 0 and 1, indicating highly favourable adsorption^33,34^.

The contact time effect was studied in the experiments, which were carried out using 10 mL (100 mg/L of BF and CV 100 mg/L), and 0.01 g of the CH adsorbent at the optimum pH for each dye. The shaker was thermostatically controlled and maintained at room temperature with a constant speed of 200 rpm. The contact times varied from 5 to 210 min.

In the current study, the kinetic studies were conducted using two kinetic models, pseudo-1st order (PFO) and pseudo-2nd order (PSO), which are shown in Eqs. (7–8), to determine the adsorption rate limits^35^.

7 $$\:\frac{1}{{\mathrm{q}}_{\mathrm{t}}}=\frac{{\mathrm{k}}_{1}}{{\mathrm{q}}_{\mathrm{e}1}\mathrm{t}}+\frac{1}{{\mathrm{q}}_{\mathrm{e}1}}\:$$

8 $$\:\frac{\mathrm{t}}{{\mathrm{q}}_{\mathrm{t}}}=\frac{1}{{\mathrm{k}}_{2}{\mathrm{q}}_{\mathrm{e}2}^{2}}+\frac{\mathrm{t}}{{\mathrm{q}}_{\mathrm{e}2}}\:$$

The adsorption efficiency for the dyes under investigation is represented by the values q_e_ (mg/g) and q_t_ (mg/g), respectively, at equilibrium and at a specific time t (min), in addition to K_1_ and K_2_, which are constants for pseudo-1st order and pseudo-2nd order, respectively^36^.

### Statistical error validity

The best fitting models cannot be identified only by using the correlation coefficient (R^2^), but can be estimated using other methods of model validity evaluation^37^. The well-fitted kinetic and isotherm models were examined using a variety of error functions, which also helped to reduce the error distribution between the values derived from theoretical model correlations and experimental data. Three error functions were used: the sum of squares error (SSE), mean square error (MSE), hybrid error, and chi-square statistic (χ2). These are presented in Eqs. (9–12)^38^.

9 $$\:{\mathrm{x}}^{2}=\:\:\sum\limits_{\mathrm{i}=1}^{\mathrm{n}}{\frac{\:\:\left({\mathrm{q}}_{{\mathrm{e}}_{\mathrm{i}}^{\mathrm{e}}}\mathrm{x}\mathrm{p}-{\mathrm{q}}_{{\mathrm{e}}_{\mathrm{i}}}\mathrm{c}\mathrm{a}\mathrm{l}\right)}{{\mathrm{q}}_{\mathrm{e}\mathrm{i}}\mathrm{c}\mathrm{a}\mathrm{l}}}^{2}\:\:$$

10 $$\:\mathrm{M}\mathrm{S}\mathrm{E}=\frac{1}{\mathrm{N}\mathrm{e}\mathrm{x}\mathrm{p}}\:\sum\limits_{\mathrm{i}=`1}^{\mathrm{n}}{\left(\:{\mathrm{q}}_{{\mathrm{e}}_{\mathrm{i}}^{\mathrm{e}}}\mathrm{x}\mathrm{p}-{\mathrm{q}}_{{\mathrm{e}}_{\mathrm{i}}}\mathrm{c}\mathrm{a}\mathrm{l}\right)}^{2}\:$$

11 $$\:\mathrm{S}\mathrm{S}\mathrm{E}=\:\sum\limits_{\mathrm{i}=1}^{\mathrm{n}}{\:\left({\mathrm{q}}_{{\mathrm{e}}_{\mathrm{i}}^{\mathrm{e}\mathrm{x}\mathrm{p}}}-{\mathrm{q}}_{{\mathrm{e}}_{\mathrm{i}}}\mathrm{c}\mathrm{a}\mathrm{l}\right)}^{2}\:\:$$

12 $$\:\mathrm{h}\mathrm{y}\mathrm{b}\mathrm{r}\mathrm{i}\mathrm{d}\mathrm{e}\:\mathrm{e}\mathrm{r}\mathrm{r}\mathrm{o}\mathrm{r}=\frac{100}{\mathrm{N}\mathrm{e}\mathrm{x}\mathrm{p}-\mathrm{N}\mathrm{p}\mathrm{a}\mathrm{r}\mathrm{a}\mathrm{m}\mathrm{e}\mathrm{t}\mathrm{e}\mathrm{r}}\:\sum\nolimits_{\mathrm{i}=1}^{\mathrm{n}}\:{\left(\frac{\:\:\left({\mathrm{q}}_{{\mathrm{e}}_{\mathrm{i}}^{\mathrm{e}}}\mathrm{x}\mathrm{p}-{\mathrm{q}}_{{\mathrm{e}}_{\mathrm{i}}}\mathrm{c}\mathrm{a}\mathrm{l}\right)}{{\mathrm{q}}_{\mathrm{e}\mathrm{i}}\mathrm{e}\mathrm{x}\mathrm{p}}\right)}^{2}\:\:\:$$

where n is the number of included observations. The subscript cal refers to theoretically calculated data, while the exp subscript represents experimental data.

### The effect of temperature and thermodynamic studies

The effect of temperature, and thermodynamic studies used a series of 100 mL stoppered bottles containing 10 mL of BF 100 mg/L, 100 mg/L of CV, and 0.01 g of CH biosorbent at varying temperatures (25–50 °C). Optimal pH 8 for BF, and pH 6 for CV were shaken for 120 min on an equilibrated shaker at a constant speed of 200 rpm. The BF and CV dyes’ residual concentrations were determined following adsorption and filtration.

Adsorption enthalpy (ΔH°), adsorption free energy (ΔG°), adsorption entropy (ΔS°), and thermodynamic equilibrium constant (Kc) were computed based on the Van’t Hoff and Gibbs equations, which are thermodynamic parameters stated in Eqs. (14), and (15)^39,40^ and were computed as follows:

13 $$\:\text{}\Delta\mathrm{G}^{\mathrm{o}}=-\mathrm{RT}\mathrm{ln}{\mathrm{K}}_{\mathrm{c}}$$

14 $$\:\mathrm{ln}{\mathrm{K}}_{\mathrm{C}}\mathrm{=}\frac{{{\Delta}\mathrm{s}}^{\mathrm{o}}}{\mathrm{R}}\mathrm{-}\frac{{{\Delta}\mathrm{H}}^{\mathrm{o}}}{\mathrm{RT}}\:\:\:\:\:\:\:\:\:\:\:\:\:\mathrm{m}$$

The values of ΔS° and ΔH° were determined using Eq. (14), where the intercept equal ΔS°/R, and the slope equal − ΔH°/R of ln K_c_ vs. 1/T. The value of a gas constant (R) is 8.314 J/mol K^41^.

### Desorption and reusability studies

The reusability was tested after CV and BF were adsorbed by CH biosorbent using a variety of eluents, including NaHCO33(0.1 M), NaHCO33 (0.5 M), Na22CO33 (0.1 M), NaOH (0.1 M), NaOH (0.5 M), KCl (0.1 M), and Ethanol (99%). The reusability was used to study the process of the CH batch method over five repeated adsorption-desorption cycles. After shaking 0.01 g of CH with 10 mL of CV and BF at 100 mg/L for two hours, the adsorbent was filtered and eluted in suitable eluents. These steps were repeated four more times. Eq. 15 was used to calculate the examined dyes desorption (D %) of CH^42^.

15 $$\mathrm{Desorption}\: \mathrm{efficiency}\:\left({\%}\right)=\frac{{{\mathrm{C}}}_{\text{{d}{e}{s}}}}{{{\mathrm{C}}}_{{\mathrm{Ads}}}}\times\:100$$

where C_des_ (mg/L) is the concentration desorbed into solution, and C_ads_ (mg/L) is the concentration originally adsorbed on the sorbent.

### Analysis of real water samples

A spiking experiment was performed by adding 100 mg/L of CV and BF into real water matrices, including seawater, wastewater, and tap water. At room temperature, 0.01 g of CH was introduced into each sample, and the pH was adjusted BF to 8, and CV to 6. The mixtures were continuously shaken for 120 min to facilitate adsorption. Afterward, the samples were centrifuged to ensure complete separation of the biosorbent. The residual concentrations of CV and BF were determined at the appropriate wavelengths with a Unicam UV 2100 UV-Visible spectrometer.

## Results and discussion

### Physicochemical studies

#### Optical images

**Fig. 1:**
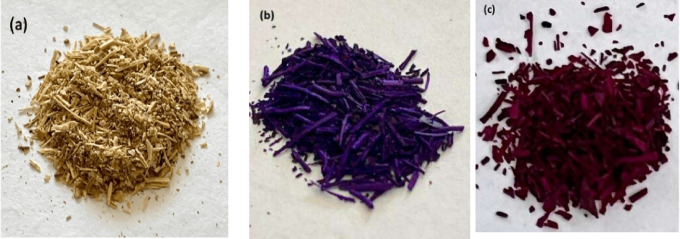
Digital photograph of **(a)** CH, **(b)** CH-CV, **(c)** CH- BF.

The optical images in Fig. 1(a-c) clearly show the gradual visual changes in CH before and after dye adsorption. Initially, as shown in Fig. 1a, the raw CH exhibits a natural beige coloration, indicative of the raw CH surface. A noticeable change in surface color is visible after the adsorption of BF and CV. The CH-CV, Fig. 1b, turns violet, whereas CH-BF, Fig. 1c, has a pink color. These color changes indicate the successful adsorption of dye molecules onto the CH biosorbent surface. The visual intensity and tone of the new colors correspond to the intrinsic properties of each dye, indicating a successful interaction with CH functional groups. This noticeable alteration highlights CH potential as a natural, inexpensive biosorbent and supports the effective binding of BF and CV onto it.

#### BET

**Fig. 2:**
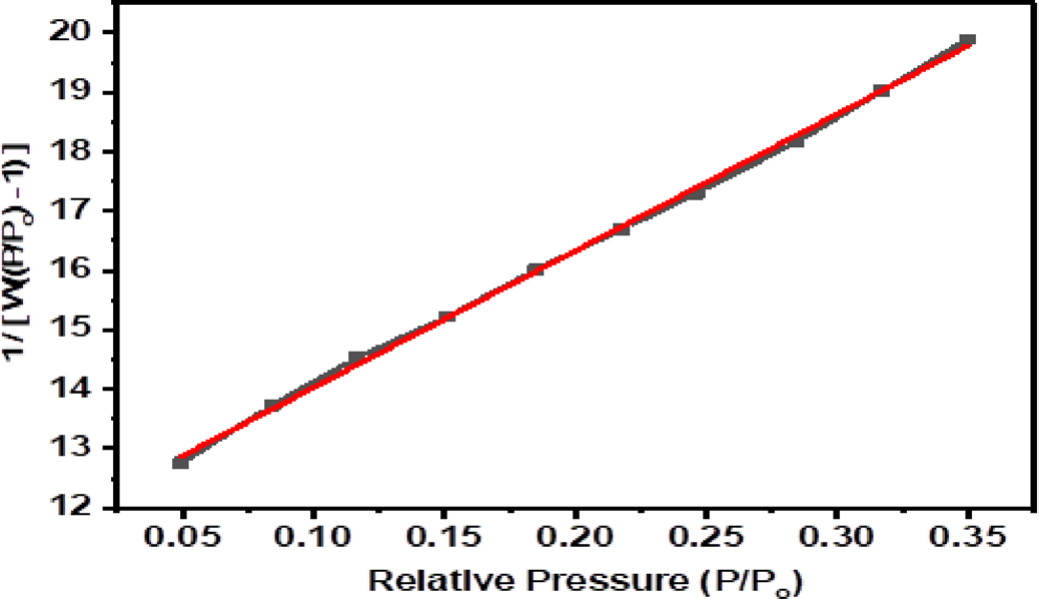
Linear BET plots of nitrogen adsorption isotherms at 77.35 °K for the CH biosorbent.

The surface area and pore textural properties of the CH biosorbent were measured with the Brunauer-Emmett-Teller (BET) method. Nitrogen adsorption–desorption measurements were performed at 77.35 K, and the data were analyzed using the linear BET equation. The linear portion of the BET plot provided a surface area of 100.249 m²/g. Figure 2; Table 3 provide additional details on nitrogen adsorption isotherms and pore structure characteristics, such as total pore volume and pore size distribution.

Table 3 shows that the average pore diameter of the CH biosorbent is 69.485 Å, significantly larger than the molecular diameters of CV(14 Å) and BF (13–18 Å), depending on the form. This indicates that the CH biosorbent pore dimensions are large enough to allow the dye molecules to diffuse into its porous structure.

**Table 3:** The textural properties of CH biosorbent.

Biosorbent	Adsorption Temp.	BET C constant	Aver. pore volume (Å)	Total pore volume (cm^3^.g^− 1^)	Surface area (m^2^.g^− 1^)
**CH**	77.35^o^K	2.9607	69.485	0.1197	100.249

### Characterization

#### Elemental analysis

The elemental analysis of CH, CH-CV, and CH- BF were investigated. As shown in Table 4, the nitrogen content increased from 0.47% in raw CH to 0.98% after CV adsorption and then slightly decreased to 0.96% after BF adsorption. The carbon percentage also increased from 43.14% to 44.80% with BF and 44.97% with CV. In contrast, the hydrogen content decreased slightly with BF from 5.46% to 5.35%, and then increased to 6.13% with CV. These changes, especially the increase in nitrogen and carbon contents, confirm the dyes’ successful adsorption on the CH surface.

**Table 4:** Elemental analysis of CH, CH-CV, and CH- BF.

Sample	C(%)	H(%)	*N*(%)
CH	43.14	5.46	0.47
CH-CV	44.97	6.13	0.98
CH- BF	44.80	5.35	0.96

#### Scanning electron microscopy

**Fig. 3:**
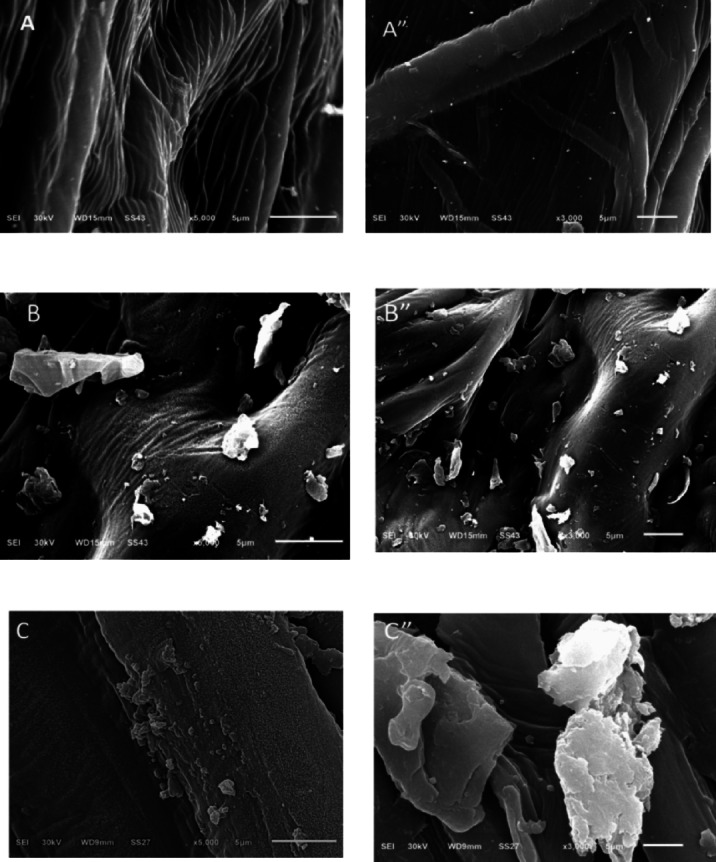
SEM photographs of **(A**,** A”)** CH biosorbent, **(B**,** B”)** CH-CV, and **(C**,** C”)** CH- BF.

The SEM images show morphological changes on the CH surface at various stages of the dye adsorption process, as shown in Fig. 3(A-C”). In Fig. 3(A, A’’), the surface appears relatively smooth and organized, with visible fibrous structures that correspond to the natural cellular architecture of plant cell walls. The surface is free of impurities, indicating a clean starting point^43^.

After CV adsorption, the CH-CV in Fig. 3(B, B’’) shows the presence of particle aggregates and uneven surface deposition, indicating that dye molecules interact effectively with the CH surface. The dye appears to settle in surface grooves, most likely due to physical or electrostatic interactions^44^. In the case of BF adsorption, the CH- BF, as shown in Fig. 3(C, C’’), the surface exhibits significant roughness and dense dye accumulation, indicating nearly complete surface coverage and possibly strong interactions between the dye and the active functional groups on the CH surface. These results confirm CH high efficiency as a low-cost and promising biosorbent for removing dyes from aqueous solutions^45^.

#### FTIR spectra

**Fig. 4:**
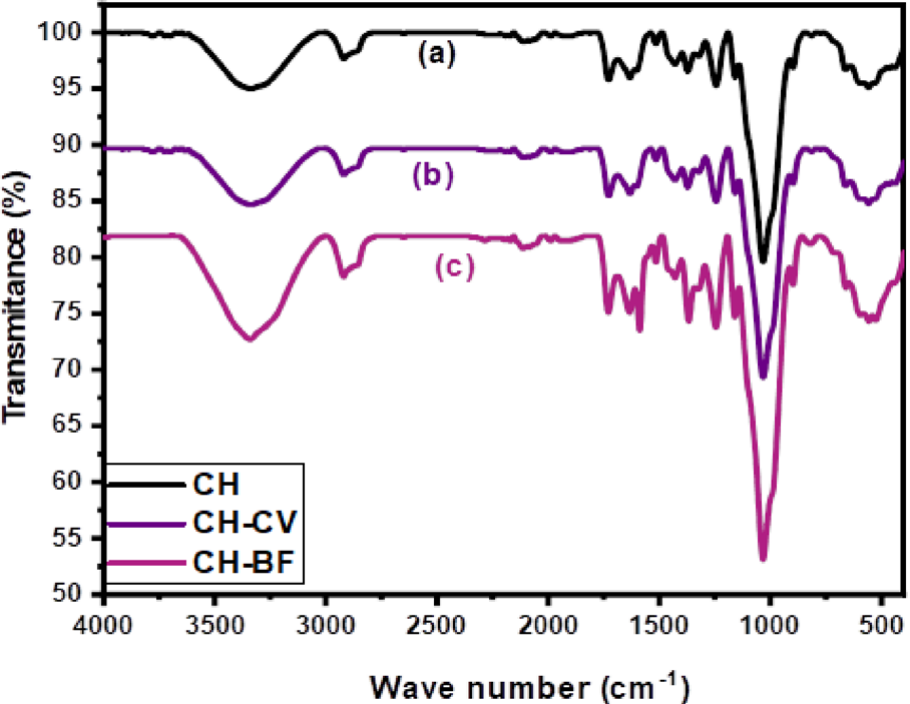
FTIR spectra of: **(a)** CH biosorbent, **(b)**CH-CV, and **(c)**CH- BF.

The FTIR spectra of CH, CH-CV, and CH- BF, shown in Fig. 4, were analyzed between 400 and 4000 cm⁻¹ to identify the functional groups involved in dye adsorption on CH biomass.

The spectrum of raw CH, as shown in Fig. 4a, displays a broad absorption band centered around 3330 cm⁻¹, corresponding to the O–H stretching vibrations of hydroxyl groups, indicative of cellulose, hemicellulose, and lignin content. A shoulder observed near 2920 cm⁻¹ is attributed to the asymmetric and symmetric stretching of aliphatic –CH₂ and –CH₃ groups. A strong and sharp band around 1026 cm⁻¹ is assigned to C-O stretching vibrations of cellulose and hemicellulose. A peak at 875–900 cm⁻¹ indicates skeletal vibrations and fingerprint region deformations in complex biopolymer matrices^46,47^.

When CV adsorption, as observed in Fig. 4b, the O-H band narrows and shifts, indicating interactions with hydroxyl and carbohydrate groups. The skeletal band shifts to approximately 865 cm⁻¹, suggesting structural changes.

On the other side, after BF adsorption, as observed in Fig. 4c, the O-H band broadens and weakens, while the C-O band intensity decreases, indicating stronger interactions. The skeletal band extends to 855 cm⁻¹.

These spectral changes confirm successful dye adsorption on CH, primarily through hydrogen bonding, electrostatic interactions, and possible π-π interactions. This highlights CH’s potential as an eco-friendly biosorbent.

#### Thermogravimetric analysis

**Fig. 5:**
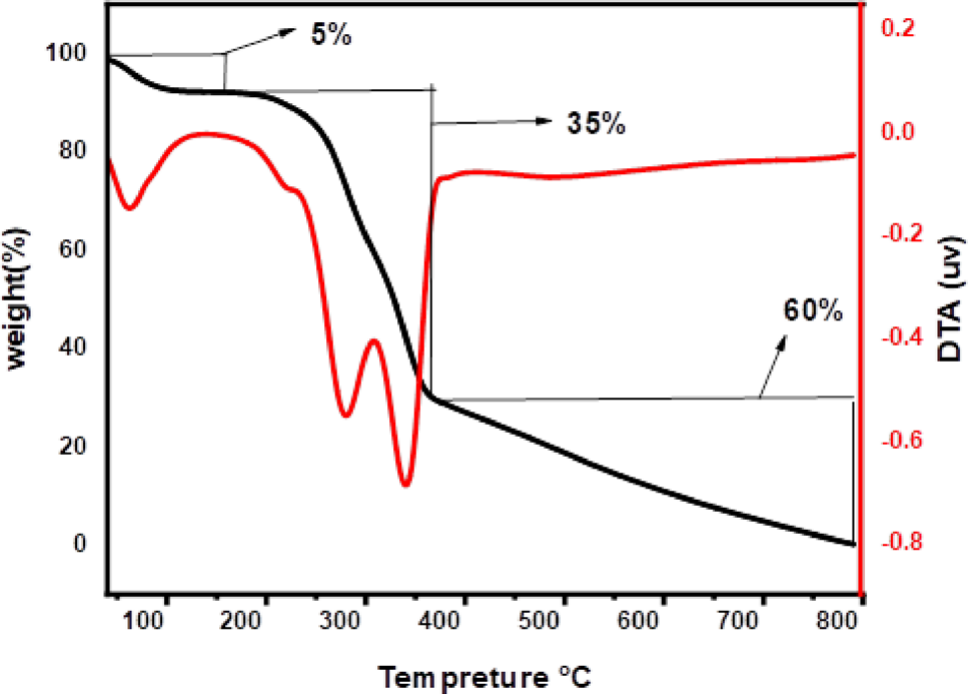
Thermal analysis of CH biosorbent.

Thermogravimetric analysis (TGA) was used to evaluate the thermal behavior of CH at temperatures ranging from 25 to 800 °C, as shown in Fig. 5. The decomposition occurred in three distinct stages, indicating the gradual thermal degradation of the lignocellulosic structure.

In the first stage (25–150 °C), surface-bound moisture evaporated, resulting in a slight weight loss of about 5%^48^. The second stage, between 150 °C and 400 °C, resulted in a weight loss of approximately 35% due to the degradation of hemicellulose and cellulose, the primary carbohydrate components in CH. The third stage, ranging from 400 °C to 800 °C, resulted in a 60% weight loss due to lignin breakdown and gradual volatilization of organic matter. This suggests that a significant portion of the biomass undergoes thermal decomposition only at high temperatures^49^.

These findings confirm that CH contains a large proportion of thermally active organic components and has a gradual thermal decomposition profile. Its moderate thermal stability and high carbon content make it potentially suitable for use in environmental remediation and thermal valorization processes.

#### Optimization of dye adsorption onto CH biosorbent using RSM

The effects of pH and initial dye concentration C_i_ on the removal efficiency of CV and Basic Fuchsin BF using CH biosorbent were systematically investigated using Response Surface Methodology RSM with experimental design carried out via Central Composite Design CCD in Design Expert version 19.

Response Surface Methodology RSM was applied to evaluate the combined effects of pH A and C_i_ B on the removal efficiencies of both dyes. The complete set of experimental runs along with the observed and predicted responses is presented in Tables (5 & 6) where excellent agreement is observed between the actual Removal percentage values and the predicted values generated by the quadratic models. For CV the maximum observed removal reached 77.1% with a predicted value of 76.09% while for BF the highest removal was 76.8% with a predicted value of 75.06%. Both dyes exhibited reduced removal under highly acidic pH equal to 2 or alkaline pH equal to 10 conditions and very low or very high dye concentrations also decreased efficiency.

The quadratic models fitted to the experimental data demonstrated strong predictive capability. For CV the model exhibited excellent Performance with R² = 0.9910, adjusted R² = 0.9865, and predicted R² = 0.9631, indicating very good agreement between experimental and predicted removal efficiencies. The adequate precision of 23.38 and a low coefficient of variation (CV%) = 6.86 confirm a strong signal-to-noise ratio and high reproducibility, validating the reliability of the RSM model for CV. For BF the RSM model also showed good predictive capability, though slightly lower than CV, with R² = 0.9604, adjusted R² = 0.9394, and predicted R² = 0.8387. The adequate precision of 11.02 indicates a strong signal relative to noise, confirming the model’s ability to accurately predict BF removal, albeit with slightly higher variability than CV.

The ANOVA results summarized in Table 7 indicate that pH A was the most significant factor influencing removal efficiency for both dyes with p less than 0.0001 while the effect of initial concentration C_i_ B was comparatively less pronounced and, in some cases, not statistically significant with p equal to 0.177 for CV and 0.643 for BF. The quadratic terms pH squared and C_i_ squared were significant for both dyes confirming the curvature of the response surfaces while the interaction term pH times C_i_ was not significant suggesting that the factors act mostly independently.

The CCD structure incorporated factorial points PtType minus 1 to assess linear effects and interactions, axial points PtType plus 1 to detect curvature, and center points PtType zero to estimate experimental error and verify reproducibility. All runs were performed within a single block Block 1 ensuring consistent experimental conditions^50^.

The final quadratic regression calculated by Eq. 3 describes the response surface for CV and BF, respectively:

$${\mathrm{Y}}\,{\mathrm{=}}\,{\mathrm{41}}{\mathrm{.925-25}}{\text{.12 A2}}\,{\mathrm{+}}\,{\mathrm{21}}{\text{.39 A6}}\,{\mathrm{+}}\,{\mathrm{3}}{\text{.73 A10-4}}{\text{.86 B50}}\,{\mathrm{+}}\,{\mathrm{12}}{\text{.78 B100-7}}{\text{.92 B150}}$$

$${\mathrm{Y}}\,{\mathrm{=}}\,{\mathrm{44}}{\mathrm{.32-26}}{\text{.52 A2}}\,{\mathrm{+}}\,{\mathrm{17}}{\text{.61 A6}}\,{\mathrm{+}}\,{\mathrm{8}}{\text{.90 A10-5}}{\text{.28 B50}}\,{\mathrm{+}}\,{\mathrm{13}}{\text{.04 B100-7}}{\text{.76 B150}}$$

**Fig. 6:**
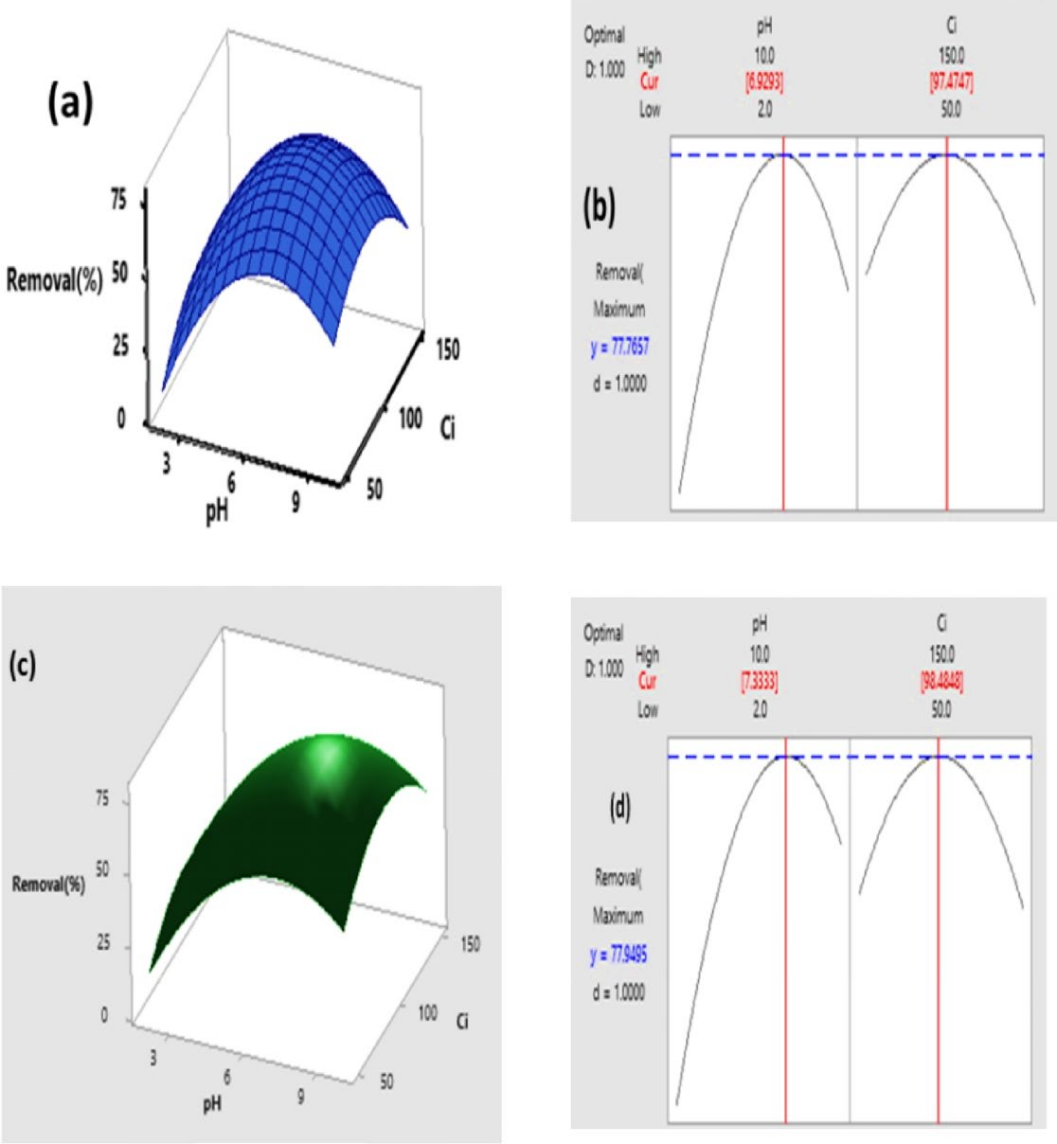
**(a)** 3D response surface showing the interactive effect of pH and initial concentration (C_i_) on removal efficiency for CV **(b)** Optimization plot indicating the optimal levels of pH and initial concentration (C_i_) for maximizing removal efficiency for CV **(c)** 3D response surface showing the interactive effect of pH and initial concentration (C_i_) on removal efficiency for BF **(d)** Optimization plot indicating the optimal levels of pH and initial concentration (C_i_) for maximizing removal efficiency for BF.

The three-dimensional response surfaces and contour plots in Fig. 6(a, c) illustrate the interactive effects of pH and C_i_. These plots demonstrate that removal efficiency increases sharply at moderate pH around 6 to 7 and intermediate dye concentrations around 100 mg per liter while extreme acidic, alkaline, or very high or low concentrations decrease efficiency. Optimization plots in Fig. 6b indicate the optimum operating conditions at approximately pH equal to 6.9 and C_i_ around 97–100 mg/L for CV with a desirability value equal to 1, and Fig. 6d for BF indicate the optimum operating conditions at approximately pH equal to 7.3 and C_i_ around 98–100 mg/L with a desirability value equal to 1.

Overall, the integration of RSM via CCD, ANOVA evaluation, model adequacy metrics, and graphical response visualization confirms that CH biosorbent is highly effective for removing both CV and BF dyes under moderate pH conditions and the developed quadratic models provide a robust and reliable tool for predicting and optimizing dye removal under a wide range of operational conditions.

**Table 5:** RSM-based experimental runs for CV removal using CH, showing pH, initial concentration, observed removal (%), and predicted values (FITS1).

Run Order	pH	C_i_	PtType	Blocks	Removal(%)	FITS1
1	6	100	0	1	77.1	76.08966
2	6	150	−1	1	51.4	55.39253
3	6	100	0	1	77.1	76.08966
4	6	100	0	1	77.1	76.08966
5	6	100	0	1	77.1	76.08966
6	2	150	1	1	13.3	11.26874
7	2	50	1	1	10.13	9.565402
8	10	150	1	1	37.32	35.35874
9	2	100	−1	1	26.99	29.58586
10	6	100	0	1	77.1	76.08966
11	10	50	1	1	43.67	43.1754
12	6	50	−1	1	57.39	58.4492
13	10	100	−1	1	55.98	58.43586

**Table 6:** RSM-based experimental runs for BF removal using CH, showing pH, initial concentration, observed Removal (%), and predicted values (FITS1).

Run Order	pH	C_i_	PtType	Blocks	Removal(%)	FITS1
1	6	150	−1	1	44.8	54.33
2	6	50	−1	1	56.9	56.69
3	6	100	0	1	76.8	75.06
4	2	150	+ 1	1	16.22	8.99
5	2	100	−1	1	20.98	30.9
6	10	50	+ 1	1	44	46.85
7	6	100	0	1	76.8	75.06
8	6	100	0	1	76.8	75.06
9	2	50	+ 1	1	16.22	13.71
10	10	150	+ 1	1	48.68	46.85
11	6	100	0	1	76.8	75.06
12	6	100	0	1	76.8	75.06
13	10	100	−1	1	67	66.4

**Table 7:** The ANOVA results of the quadratic models.

Dyes	Source	DF	Adj SS	Adj MS	F-Value	*P*-Value
**CV**	**Model**	5	7310.53	1462.11	235.43	0.000
	**Linear**	2	1262.50	631.25	101.65	0.000
	**pH**	1	1248.48	1248.48	201.04	0.000
	**C**_**i**_	1	14.01	14.01	2.26	0.177
	**Square**	2	6025.37	3012.69	485.11	0.000
	pH*pH	1	2842.14	2842.14	457.65	0.000
	Ci*Ci	1	1014.84	1014.84	163.41	0.000
	pH*Ci	1	22.66	22.66	3.65	0.098
	**Error**	7	43.47	6.21		
	**Total**	12	7354.00			
	**R-Squared**	0.9910				
	**Adjusted R-Squared**	0.9865				
	**Predicted R-Squared**	0.9631				
	**Adequate Precision**	23.38				
BF	**Model**	5	6659.07	1331.81	33.94	0.000
	**Linear**	2	1891.04	945.52	24.10	0.001
	**pH**	1	1881.86	1881.86	47.96	0.000
	**C**_**i**_	1	9.18	9.18	0.23	0.643
	**Square**	2	4762.56	2381.28	60.69	0.000
	pH*pH	1	1927.60	1927.60	49.13	0.000
	Ci*Ci	1	1056.50	1056.50	26.93	0.001
	pH*Ci	1	5.48	5.48	0.14	0.720
	**Error**	7	274.67	39.24	-	-
	**Total**	12	6933.74	-	-	-
	**R-Squared**	0.9604				
	**Adjusted R-Squared**	0.9394				
	**Predicted R-Squared**	0.8387				
	**Adequate Precision**	11.02				

### Adsorption studies

#### Point of zero charge and pH effect

The point of zero charge (PZC) is an essential parameter in characterizing biosorbents and provides valuable insight into the electrostatic interactions between the biosorbent surface and the sorbate. As illustrated in Fig.S2, the point of zero charge (pH_final_ - pH_initial_) of the CH biosorbent was determined to be 4.6, based on the variation between initial and final pH values as a function of initial pH. This result indicates that the CH surface is positively charged at pH values below 4.6 and becomes negatively charged above this value.

#### Effect of CH biosorbent dosage

**Fig. 7:**
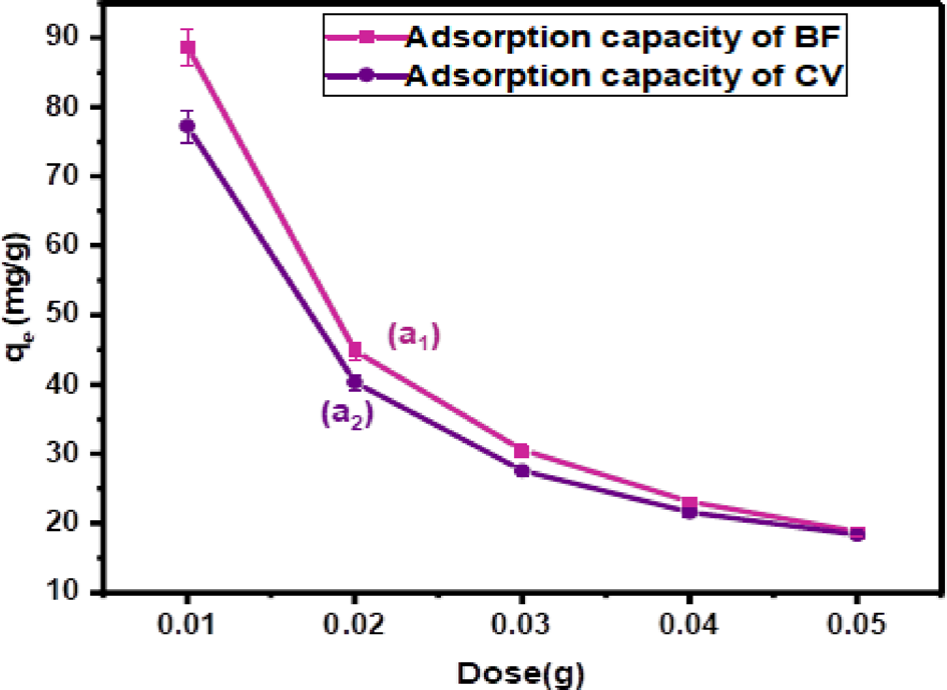
Effect of dose on the adsorption **(a**_**1**_**)** of CV on CH of these conditions (temp.: (25 °C), pH = 6, conc.: 100 mg/L, volume: 10 mL,120 min), and **(a**_**2**_**)** on the adsorption of BF on CH (temp.: (25 °C), pH = 8, conc.: 100 mg/L, volume: 10 mL,120 min).

The effect of CH biosorbent dosage on dye adsorption was systematically investigated by varying its mass in 10 mL solutions containing 100 mg/L of cationic dyes BF and CV, with an agitation time of 120 min at room temperature, as shown in Fig. 7(a_1_–a_2_). As illustrated in Fig. 7a_1_, increasing the CH dosage from 0.01 g to 0.05 g resulted in a significant reduction in the adsorption capacity of BF at pH = 8, decreasing from 88.8 mg/g to 18.8 mg/g. A similar trend was observed in Fig. 7a_2_ for CV at pH = 6, where the adsorption capacity dropped from 77.3 mg/g to 18.34 mg/g with the same increase in CH dosage. The decrease in adsorption capacity per gram of CH biosorbent at higher dosages can be attributed to an excess of active sites compared to the number of dye molecules, resulting in underutilization. Furthermore, at high dosages, particle aggregation may reduce effective surface area and prevent dye access to certain adsorption sites. This behavior is commonly observed in batch adsorption systems.

#### Effect of initial dye concentration and adsorption isotherms

**Fig. 8:**
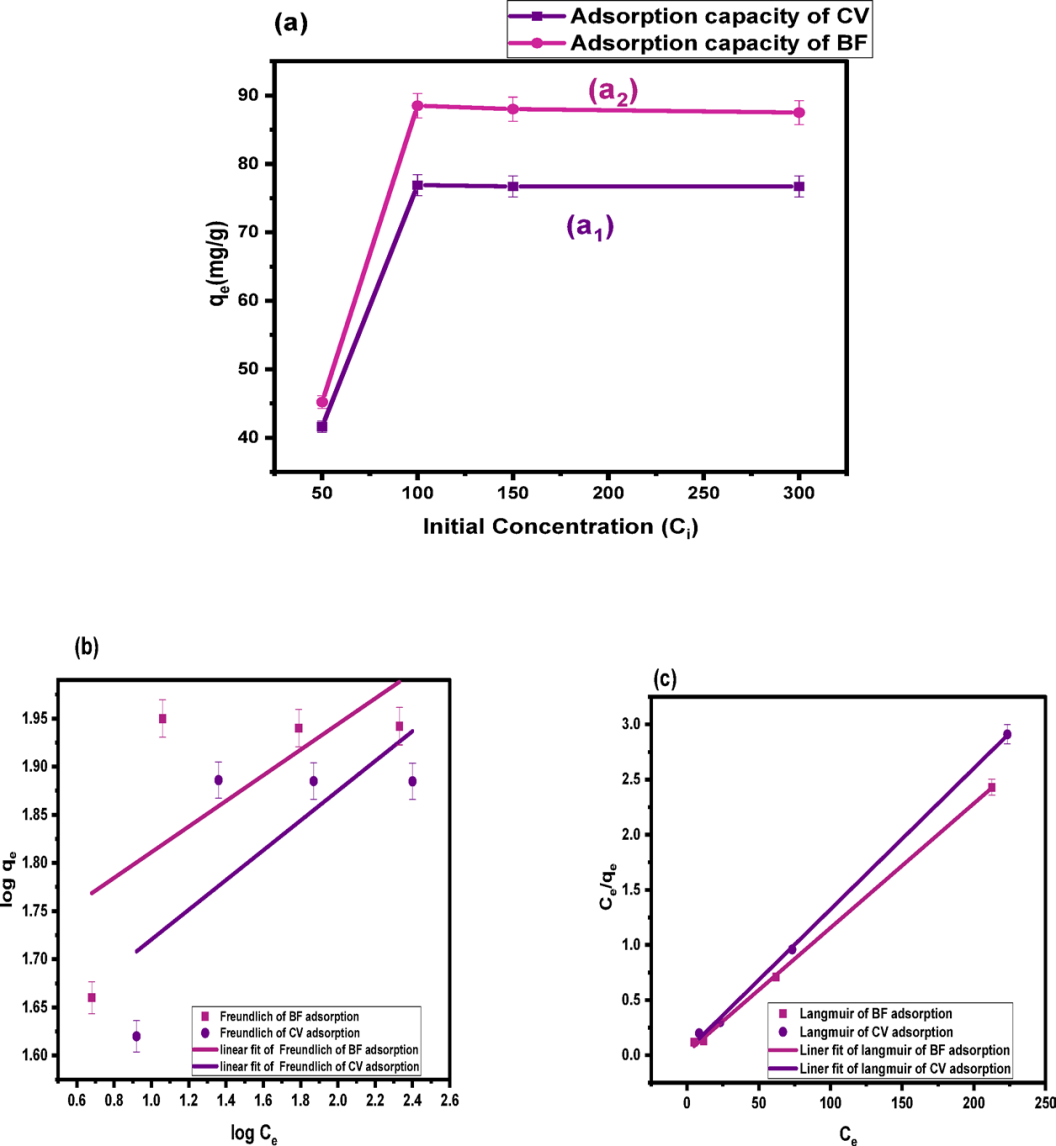
**(a) **Effect of concentration of BF (pH = 8, temp.: (25 °C), dose: 0.01 g, volume: 10 mL, 120 min), and CV (pH = 6, temp.: (25 °C), dose: 0.01 g, volume: 10 mL, 120 min), **(b)** linear Freundlich for BF, and CV adsorption, and **(c)** linear Langmuir for BF, and CV adsorption.

Batch adsorption experiments were conducted to evaluate the effect of initial dye concentration (50–300 mg/L) on the uptake of CV and BF by CH biosorbent, under constant temperature, adsorbent dosage, and optimal pH. As shown in Fig. 8(a_1_–a_2_), qe increased from 41.6 to 76.9 mg/g for CV and from 45.2 to 88.5 mg/g for BF up to 100 mg/L, beyond which adsorption reached equilibrium due to site saturation. This reflects the high affinity of CH biosorbent at moderate concentrations.

The adsorption mechanism was further examined using the linear forms of Langmuir and Freundlich isotherms Fig. 8(b–c). Model parameters (q_m_, n, K_L_, K_F_) were applied Eqs. (4) and (5) and error analyses (χ², SSE, MSE, HYBRID) are summarized in Table 8. The Langmuir model provided the best fit, with R² = 0.99906 for CV and 0.99929 for BF, and predicted q_e_ values 85.69 mg/g and 77.21 mg/g, respectively, closely matching the experimental data. This confirms monolayer adsorption on a homogeneous surface with uniform energies. Conversely, the Freundlich model showed lower accuracy (R² = 0.55533 for CV and 0.47804 for BF) and higher error values, indicating surface heterogeneity.

Overall, the Langmuir isotherm successfully described the adsorption of CV at pH 6 and BF at pH 8 onto CH, confirming its effectiveness and eco-friendly potential as a dye adsorbent. The dimensionless separation factor (R_L_), calculated from Eq. (6), was 0.652 for CV and 0.1626 for BF; since both values are less than 1, the adsorption process was demonstrated to be favourable.

**Table 8:** Adsorption isotherm parameters of CV and BF by CH biosorbent.

Model	Parameters	CH-CV	CH- BF
**Langmuir**	**q**_**m**_ **(mg/g)**	78.13	88.65
	**K**_**L**_ **(min** ^**− 1**^ **)**	0.023	0.0412
	**R** ^**2**^	0.9906	0.9929
	**R**_**L**_	0.652	0.16
	**χ2**	17.152	71.28
	**SSE**	1340.07	1579.80
	**MSE**	335.0181	6319.23
	**Hybrid error**	7.31	26.77
**Freundlich**	**n**	6.5	7.514
	**K**_**F**_ **(min** ^**− 1**^ **)**	36.81	47.691
	**R** ^**2**^	0.5553	0.4780
	**χ2**	25.38	27.25978
	**SSE**	1438.35	1819.59
	**MSE**	359.58	454.8975
	**Hybrid error**	14.914	13.59926

#### Effect of contact time and kinetics investigation

**Fig. 9:**
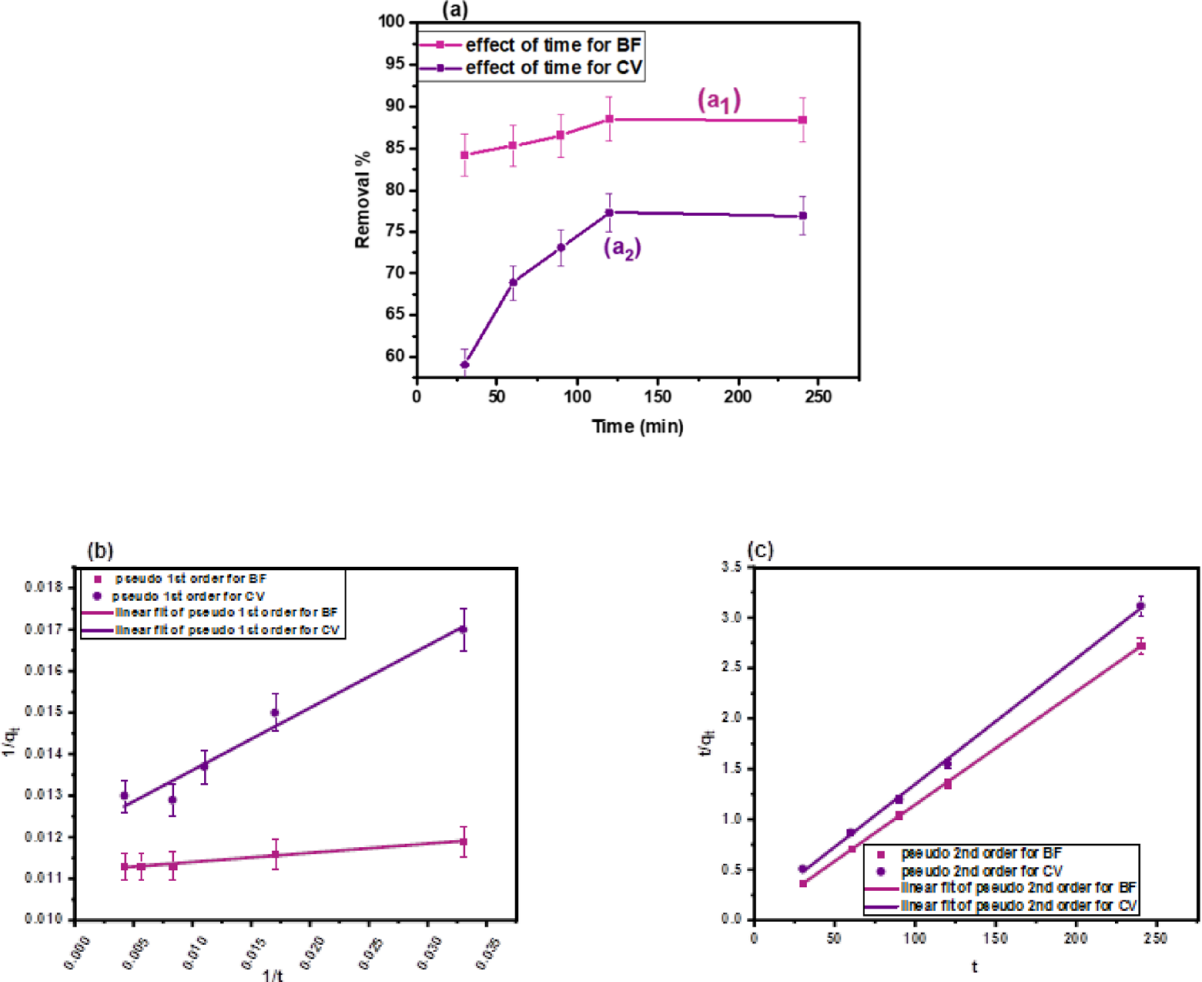
**(a)** Effect of time of **(a**_**1**_**)** BF (pH = 8, temp.: (25 °C), dose: 0.01 g, volume: 10 mL, 100 mg/L), and **(a**_**2**_**)**CV (pH = 6, temp.: (25 °C), dose: 0.01 g, volume: 10 mL, 100 mg/L), **(b)** linear PFO for BF, and CV adsorption, and **(c)** linear PSO for BF, and CV adsorption.

The effect of contact time on the adsorption efficiency of BF and CV dyes is shown in Fig. 9a. For BF Fig. 9a_1_, removal efficiency increased from 84.2% to 88.41% as contact time extended from 30 to 120 min, after which no significant change was observed, indicating equilibrium at 120 min. A similar trend was observed for CV Fig. 9a_2_, where removal efficiency rose from 59.1% to 77.3% between 5 and 120 min, then stabilized, confirming equilibrium at 120 min.

To evaluate the adsorption kinetics, the linear pseudo-first-order (PFO) and pseudo-second-order (PSO) models, Eqs. (7) and (8) were applied, and their kinetic parameters (k₁, k₂, qₑ₁, qₑ₂, R²) were estimated from Fig. 9(b–c) and summarized in Table 9. Although the PFO model Fig. 9b yielded acceptable R² values 0.98014 for BF and 0.96694 for CV, deviations between calculated and experimental q_e_ indicated a poor representation of the actual mechanism.

In contrast, the PSO model Fig. 9c exhibited excellent agreement, with higher R² values and strong consistency between calculated and experimental adsorption capacities. These results confirm that the PSO model best describes the adsorption kinetics of both dyes, suggesting that the process is primarily governed by chemisorption involving valence forces through electron sharing or exchange between the dye molecules and the CH.

**Table 9:** Kinetic parameters for the BF and CV adsorption onto CH biosorbent.

Model	Parameters	CH- BF	CH-CV
**Linear PFO**	**q**_**e1**_ **(mg/g)**	45.15	6.64
	**k**_**1**_ **(min**^**− 1**^**)**	0.5328	0.0803
	**R** ^**2**^	0.98014	0.9669
	**χ2**	190.17	3158.83
	**SSE**	8586.15	20974.59
	**MSE**	1717.23	4194.92
	**Hybrid error**	3.96	11.68
**Linear PSO**	**q**_**e2**_ **(mg/g)**	89.13	78.99
	**k**_**2**_ **(min**^**− 1**^**)**	0.448	0.00122
	**R** ^**2**^	0.9994	0.99906
	**χ2**	0.528	6.828
	**SSE**	46.99	539.34
	**MSE**	9.40	107.87
	**Hybrid error**	0.0221	0.350

#### Thermodynamic studies

**Fig. 10:**
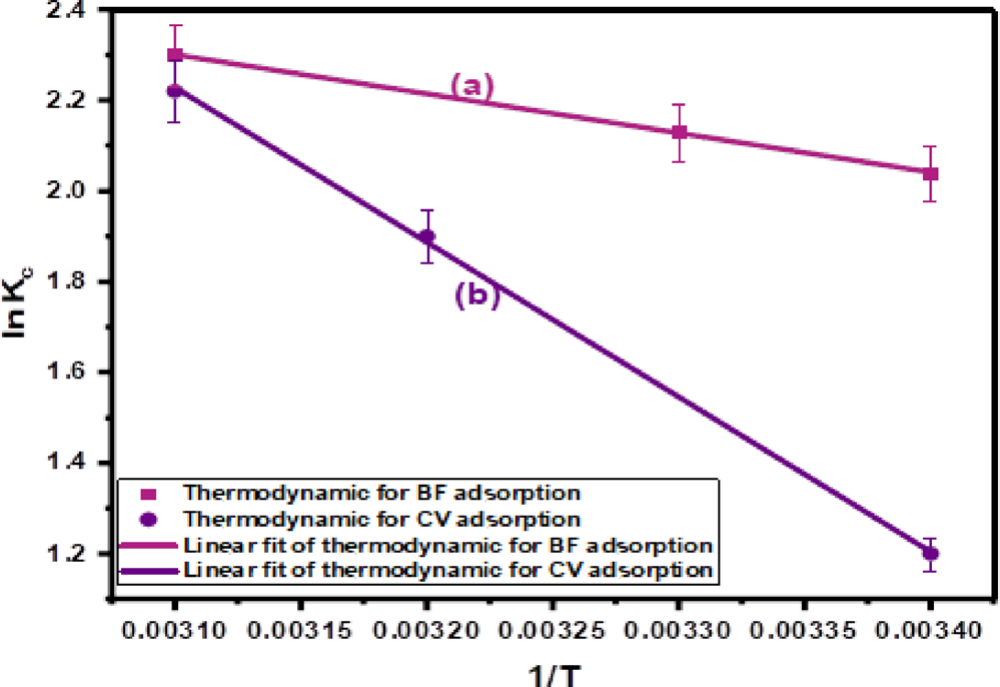
Plot of ln K_C_ versus (1/T) absolute temperature for the adsorption of (lnK_c_ vs. 1/T) for the adsorption of **(a)** BF, and **(b)**CV dye onto CH biosorbent.

The thermodynamic behavior of cationic dye adsorption, specifically CV and BF, onto the CH biosorbent was investigated over a temperature range of 298 to 323 K. This assessment enabled the determination of key thermodynamic parameters: ΔG°, ΔH°, and ΔS°. The Kc was computed based on the Vant Hoff and Gibbs Eqs. (13) and (14), and the derived values are presented in Table 10 and illustrated in Fig. 10.

The negative Gibbs free energy values (ΔG°) observed for both CV and BF across all studied temperatures (298–323 ^o^K) confirm the thermodynamic feasibility and spontaneous nature of the adsorption processes. In the CV and BF, the ΔG° values become more negative with increasing temperature, indicating that higher temperatures enhance dye adsorption and support the endothermic nature of the interaction.

This endothermic nature is clearly reflected in the positive (ΔH°) for both dyes. The calculated ΔH° for CV is + 28.39 kJ/mol, while for BF, it is + 7.18 kJ/mol. Although both values are positive (indicating energy absorption), the magnitude of ΔH° for CV is significantly higher, suggesting stronger interactions—possibly approaching the range of chemisorption. In contrast, BF adsorption may involve weaker interactions, leaning towards physisorption.

The positive (ΔS°) values provide additional evidence supporting these findings. For CV, ΔS° is 106.53 J/mol·K, while for BF it is 41.4 J/mol.K. These positive values reflect an increase in randomness at the solid–liquid interface during dye adsorption. This entropy gain is attributed to the disruption of the structured hydration shell surrounding dye molecules as they associate with the CH surface. The release of ordered water molecules and increased freedom of movement contribute to a more disordered system, supporting the overall spontaneity of the process, particularly as temperature rises.

**Table 10:** Thermodynamic parameters for the adsorption of BF and CV onto CH.

System	T (^o^K)	K_C_	∆G^o^ kJ/mol)	∆H^o^_ads_ (kJ/mol)	∆S^o^ (J/mol.K)
**CH-CV**	298	3.4	−0.45	28.39	106.53
	308	6.52	−1.62		
	323	9.2	−2.14		
**CH- BF**	298	7.7	−5.06	7.18	41.4
	308	8.43	−5.46		
	323	9.9	−6.16		

#### Effect of ionic strength

Due to the high concentration of various solutes in industrial wastewater, ionic strength plays a critical role in adsorption processes. In this study, the influence of ionic strength was evaluated using different 0.1 M electrolytes, including NaCl, Na₂CO₃, NaHCO₃, NaNO₃, and KCl. As shown in Fig. 3Sa, all anions influenced the adsorption of BF and CV onto the CH surface, with Na₂CO₃ (0.1 M) exhibiting the most notable enhancement for both dyes.

Further analysis presented in Fig. 3Sb demonstrates that increasing the Na₂CO₃ concentration from 0.05 M to 0.1 M resulted in improved adsorption capacities from 85.74 to 90.87 mg/g for BF, and 62.2 to 68.34 mg/g for CV. Beyond 0.1 M, the adsorption capacities plateaued. This enhancement is attributed to the alkaline nature of Na₂CO₃, which increases the solution pH and induces deprotonation of surface functional groups, thus increasing the negative charge on the CH surface. The enhanced negative surface charge strengthens electrostatic attraction with the positively charged dye molecules, leading to greater adsorption efficiency.

This improvement in adsorption capacity is primarily due to the alkaline nature of sodium carbonate (Na₂CO₃), which raises the pH of the solution and promotes the deprotonation of surface functional groups on the CH adsorbent, such as -OH and -COOH. This improves adsorption performance by giving the surface a stronger negative charge, which increases electrostatic attraction to the positively charged dye molecules (CV and BF).

Furthermore, an electrical double layer may form on the adsorbent surface as a result of the solution’s high concentrations of sodium ions (Na⁺) and carbonate ions (CO₃²⁻). By stabilizing the cationic dye molecules near the CH surface and reducing electrostatic repulsion between them, this layer facilitates more efficient and sustainable dye adsorption.

#### Desorption and reusability

**Fig. 11:**
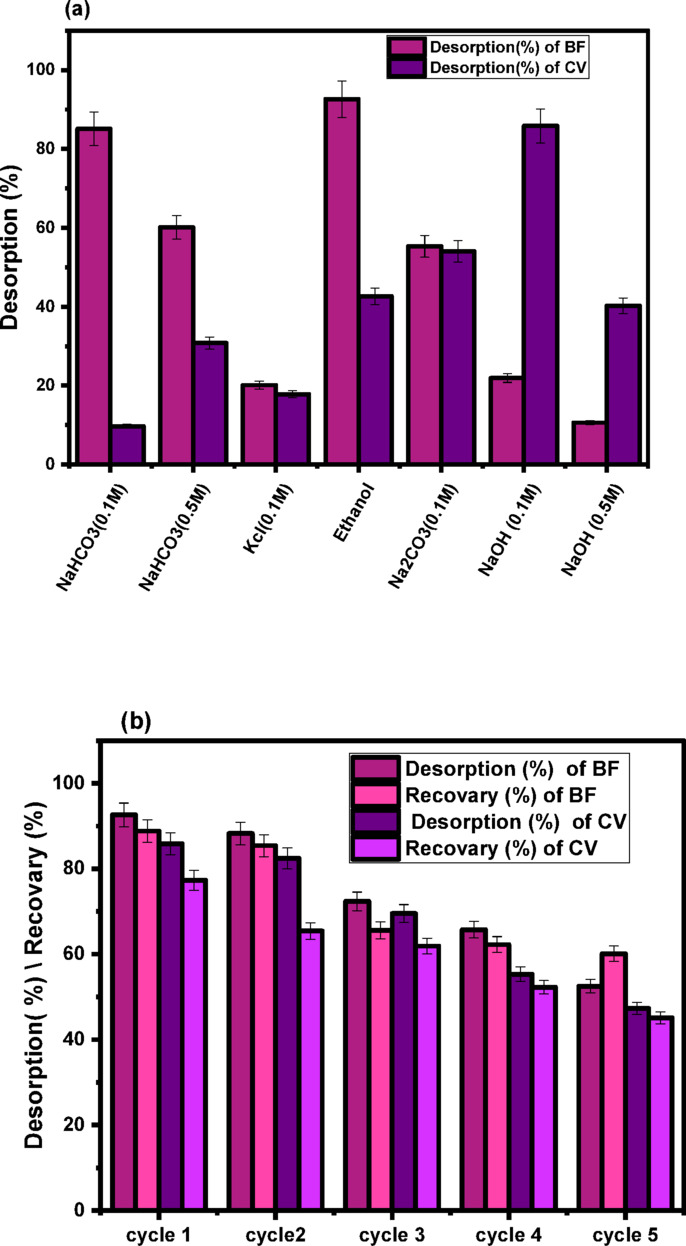
**(a)** Desorption of CV and BF from CH biosorbent by different eluents,**(b)** Repeated 5 cycles of CV and BF adsorption–desorption using Ethanol to BF and NaOH (0.1 M) to CV as eluents, (*n* = 5) *RSD relative standard deviation.

The reusability of the CH biosorbent was evaluated over five successive adsorption-desorption cycles using NaOH (0.1 M and 0.5 M), NaHCO₃ (0.1 M and 0.5 M), Na₂CO₃ (0.1 M), and Ethanol, and the desorption efficiency was calculated using Eq. (15) as shown in Fig. 11a. Ethanol was most effective for BF because it disrupted π-π stacking and hydrophobic interactions. NaOH at 0.1 M was the most efficient for CV because it promoted surface deprotonation and enhanced electrostatic repulsion.

Although 0.5 M NaOH facilitated dye desorption, its higher alkalinity may cause partial degradation or structural changes to the CH surface, making 0.1 M the more preferable and sustainable choice. After five cycles, CH retained over 90% of its initial adsorption capacity for BF with Ethanol and over 80% for CV with NaOH (0.1 M), demonstrating their suitability for repeated.

#### Removal of BF and CV in multi-contaminant systems

**Fig. 12:**
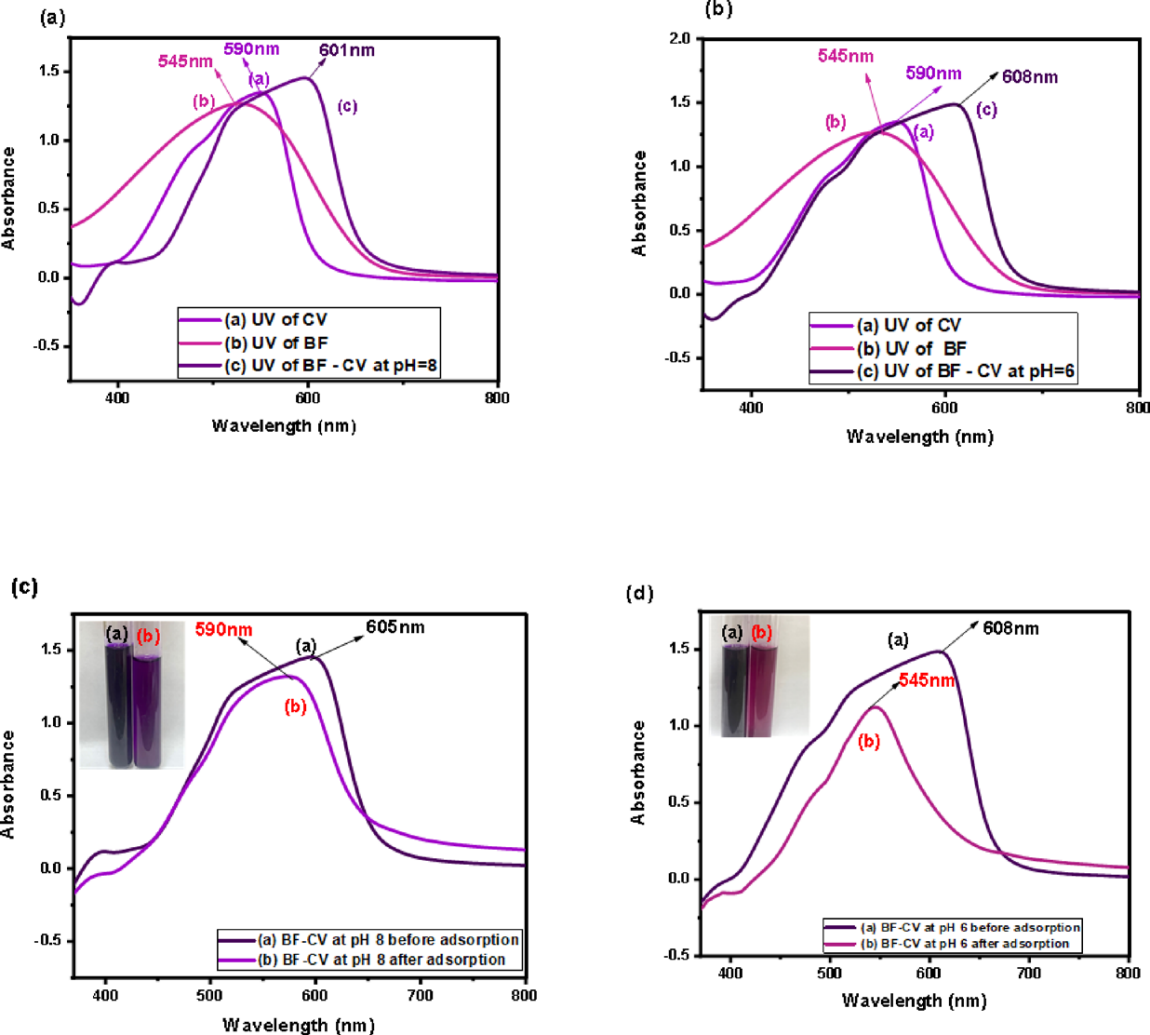
UV spectra of (**a)** BF -CV mixture at pH = 8 is compared with BF and CV, **(b)** CV- BF mixture at pH = 6 is compared with CV and BF, **(c)** CV- BF mixture at pH = 8, **(d)** CV- BF mixture at pH = 6 after adsorption by CH biosorbent.

Figure 12**(a–b)** demonstrates that, for the binary mixture of CV and BF, new absorption bands emerged close to the λ_max_ of the individual dyes (545 nm for CV and 590 nm for BF). Specifically, overlapped peaks were observed at 608 nm at pH 6 and at 601 nm at pH 8, confirming the coexistence of both dyes in the system.

Moreover, Fig. 12(c–d) reveals that the λ_max_ of the binary mixture was positioned between those of the single dyes, indicating the simultaneous adsorption of CV and BF without the formation of intermediate species. At pH 6, the preferential adsorption of CV shifted the solution color from dark purple to pink, accompanied by a λ_max_ shift to 608 nm. Conversely, at pH 8, the preferential adsorption of BF caused the solution color to change from dark purple to violet, with a corresponding λ_max_ shift to 605 nm. This pH-dependent selectivity highlights the adaptive nature of the adsorbent, whereby the dominant dye removal is dictated by the solution pH. The observed tunable adsorption behavior demonstrates the capability of the prepared adsorbent for the effective removal of cationic dye mixtures from wastewater.

#### Analysis of cationic dyes in real water samples

The applicability of the CH biosorbent for removing the cationic dyes CV and BF was evaluated using real environmental water samples representing various sources, including seawater, tap water, and wastewater. All experiments were conducted under previously optimized conditions. Calibration curves were established using standard dye solutions to ensure analytical accuracy.

Two types of real samples were tested: tap water collected from the laboratory at Mansoura University, and both wastewater and seawater obtained from Damietta City. As shown in Table 11.

The results demonstrated excellent recovery rates, ranging from 90.5% to 98.01% for BF and 92.6% to 99.1% for CV, with relative standard deviations (RSD) generally below 1.5%, indicating high precision and reproducibility. These findings highlight the potential of CH as an eco-friendly, effective biosorbent suitable not only for controlled laboratory conditions but also for realistic environmental applications involving dye-contaminated water.

**Table 11:** Analytical results of adsorption of CV and BF dyes in real water samples utilizing CH biosorbent (*n* = 5) *RSD relative standard deviation.

Sample	Dyes	Spiked (µg. mL^− 1^)	Measured (µg. mL^− 1^)	Recovered (µgmL^− 1^)	Recovery (%)	RSD (%)
Sea water	BF	0.00	0.00	0.00	0.00%	-
		50	7.7	42.3	93.8%	0.87
		100	3.67	86.33	98.01%	0.51
Tap water	BF	0.00	0.00	0.00	0.00%	-
		50	9.1	40.9	90.5%	1.1
		100	18.78	81.22	92.3%	0.78
Waste water	BF	0.00	0.00	0.00	0.00%	-
		50	8.11	41.89	92.09%	0.63
		100	15.87	84.13	95.6%	1.34
Sea water	CV	0.00	0.00	0.00	0.00%	-
		50	11.5	38.5	92.6%	1.45
		100	29.8	70.2	92.82%	0.98
Tap water	CV	0.00	0.00	0.00	0.00%	-
		50	9.12	40.88	98.3%	1.2
		100	23.37	76.63	99.1%	1.35
Wastewater	CV	0.00	0.00	0.00	0.00%	-
		50	10.55	39.45	94.8%	0.54
		100	25.3	74.7	96.6%	1.01

#### Performance of the CH biosorbent

In recent years, numerous biosorbents have been studied for their efficacy in adsorbing cationic dyes, such as BF and CV, from wastewater. In this regard, the current study suggests a cost-effective, sustainable, and environmentally friendly alternative.

The CH biosorbent possesses several distinguishing characteristics that make it an excellent candidate for dye removal. Its inherent functional groups, including hydroxyl and carboxyl groups, allow for efficient interaction with dye molecules through mechanisms such as electrostatic attraction, π-π stacking, n-π interactions, hydrogen bonding, and intra-particle diffusion. Furthermore, its porous structure provides a large surface area, which increases its adsorption capacity.

Aside from its functional performance, CH is readily available, biodegradable, and inexpensive, making it a viable and environmentally friendly alternative to conventional synthetic adsorbents. These characteristics highlight its potential as a sustainable solution for treating dye-contaminated industrial effluents. Table 12 provides a comparative overview of its adsorption performance relative to other reported materials, highlighting the CH ability to remove BF and CV.

**Table 12:** Comparison of the maximum sorption capacity of CV and BF by the proposed CH biosorbent with some of the previously published articles.

Adsorbent	q_e_ (mg/g)	Dye	Ref.
Corn husk–derived silica xerogel	69.44	CV	^51^
Beet pulp shreds	28.07	CV	^52^
Pepper Seed	82.24	CV	^53^
Pristine Corn Husk Biosorbent	77.3	CV	This study
Composite of chitosan with montmorillonite (CS-MMT)	53.11	BF	^54^
hydrolyzed soybean straw	72.9	BF	^55^
Activated carbon from pineapple crown residue (PCAC)	171.5	BF	^56^
Pristine Corn Husk Biosorbent	88.8 mg/g	BF	This study

#### Plausible mechanism of adsorption of CV and BF onto CH biosorbent

**Fig. 13:**
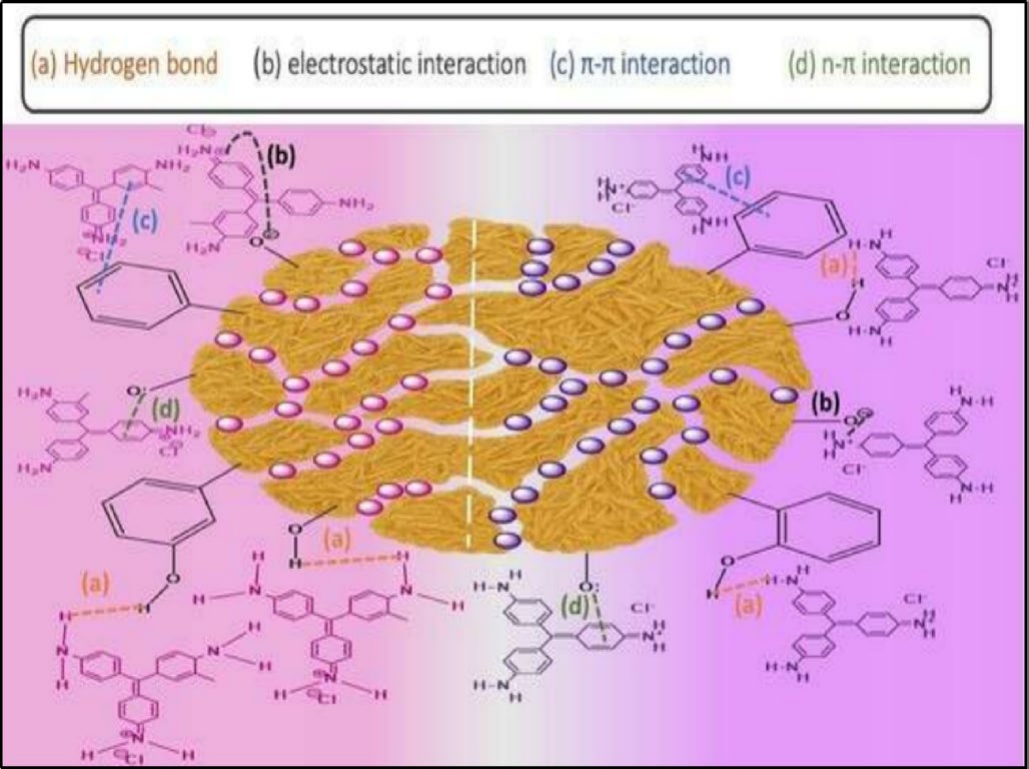
Schematic illustration of BF and CV dyes adsorption on the CH biosorbent surface.

The adsorption of dye molecules onto the CH biosorbent surface involves different simultaneous mechanisms. Firstly, hydrogen bonding is formed between hydroxyl (–OH) and amino (–NH) groups of the CH biosorbent and the electronegative atoms (O or N) of the dye molecules, which enhances molecular stability. Secondly, electrostatic interactions occur between the negatively charged functional groups on the CH biosorbent surface (e.g., –COO⁻) and the positively charged centers of cationic dyes, thereby promoting a strong attraction. In addition, π–π- stacking interactions take place between the aromatic rings of the dye molecules and the aromatic domains of the lignocellulosic components of the CH biosorbent, which further stabilize the adsorption process. Moreover, n–π interactions are established between lone pair electrons of heteroatoms such as O or N on the CH biosorbent surface and the π-electron cloud of aromatic dye rings. Altogether, these cooperative mechanisms ensure efficient and stable dye removal, which explains the high adsorption capacity of the CH biosorbent, as shown in Fig. 13.

### Future perspectives, advantages, and limitations

#### Future perspectives

The current study demonstrated the efficiency of the CH biosorbent in removing both cationic CV and BF dyes. However, further investigations are recommended to:

- Explore the adsorption behavior of CH in multi-component dye systems that better simulate real wastewater.
- Apply error function analysis to adsorption isotherm models for a more accurate evaluation of model fitting.
- Extend the application to actual wastewater samples beyond synthetic solutions.

Investigate the reusability and long-term stability of adsorbents under continuous operation.

#### Advantages of the current study

Effective Removal of both cationic CV and BF dyes, detailed characterization of CH before and after adsorption, high adsorption capacity due to multiple functional groups, the use of an abundant, low-cost agricultural biosorbent, and potential application in real wastewater treatment.

#### Limitations of the current study

Lack of column adsorption experiments, which are essential for scale-up, regeneration experiments are limited to batch cycles requiring further long-term reusability studies and the lack of biodegradability or stability assessment under real environmental conditions.

## Conclusion

**Fig. 14:**
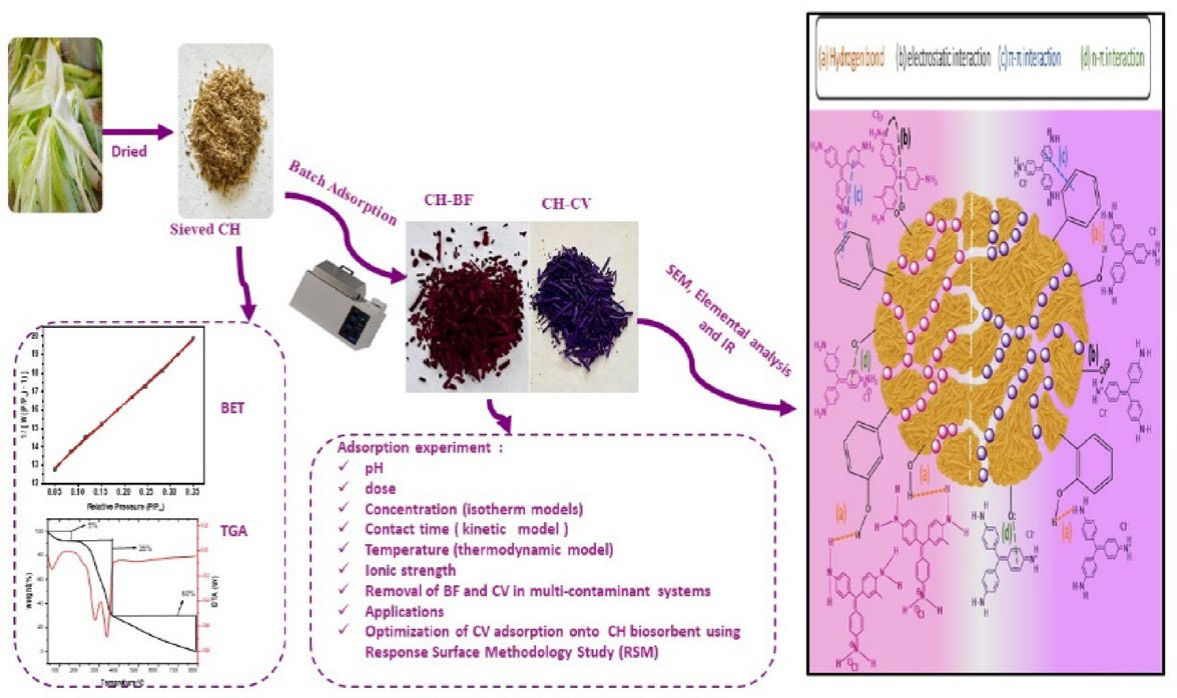
Synthesis, characterization, adsorption experiments parameters and adsorption mechanism.

In this study, the CH biosorbent was demonstrated to be a highly efficient and sustainable material for the adsorption of BF and CV dyes from aqueous media. The CH biosorbent achieved remarkable adsorption capacities of 77.3 mg/g for BF and 88.8 mg/g for CV under optimal conditions. Operational parameters, including contact time, initial dye concentration, CH dosage, solution pH, and temperature, strongly influenced the adsorption efficiency. Kinetic and equilibrium analyses revealed that the adsorption of both dyes followed the pseudo-second-order model and the Langmuir isotherm, with R² values ≥ 0.999 and low error function values, confirming that the adsorption is chemisorption in a monolayer pattern. Thermodynamic studies further indicated that the process is spontaneous and endothermic, as confirmed by the negative ΔG° values and positive ΔH° values. Unlike earlier research focusing on single-pollutant systems, this work highlights the versatility of CH biosorbent in efficiently removing more than one hazardous dye from aqueous solutions and real water samples, with recovery exceeding 90%. The CCD-based RSM model demonstrated excellent predictive accuracy and statistical validity, successfully identifying the optimal pH and initial dyes concentration for maximizing removal efficiency, thereby confirming its reliability as a robust tool for process optimization. The CH biosorbent also displayed excellent regeneration and structural stability, maintaining a removal efficiency of more than 85% after five cycles of adsorption–desorption. The proposed adsorption mechanism involves electrostatic interactions, π–π stacking, n–π interactions, hydrogen bonding, and pore diffusion. Overall, this study provides valuable mechanistic insights into the adsorption of BF and CV by the CH biosorbent, offering guidance for future wastewater treatment applications. Importantly, the valorization of CH as an eco-friendly adsorbent not only contributes to effective dye removal but also promotes environmental sustainability by reducing agricultural waste and minimizing ecological risks associated with dye pollutants. The synthesis, characterization, adsorption experiments parameters and adsorption mechanism are graphically represented in Fig. 14.

## Supplementary Information

Below is the link to the electronic supplementary material.

Supplementary Material 1

## Data Availability

Data is provided within the manuscript or supplementary information files.
