# Supplementary material for: Mechanistic insights and process optimization of pristine corn husk biosorbent for sustainable and cost effective removal of cationic dyes from waste water
Source: Sci Rep. 2026 Apr 13;16:12220. doi: 10.1038/s41598-026-45206-9 (PMC13077001; doi:10.1038/s41598-026-45206-9)
Supplement: Supplementary file 1 — Supplementary Material 1 [file 41598_2026_45206_MOESM1_ESM.docx]

**Supplementary data**

**Mechanistic Insights and Process Optimization of Pristine Corn Husk Biosorbent for Sustainable Removal of Cationic Dyes from Wastewater**

**Magda A Akl^1^*, Aya G Mostafa^1^, Asmaa A Serage^1^ and Noha A Abd-Rabo^1^**

^1^Department of Chemistry, Faculty of Science, Mansoura University, Mansoura 35516, Egypt

***Corresponding author**

**Prof Magda A Akl**

**email: [magdaakl@yahoo.com](mailto:magdaakl@yahoo.com)**

| 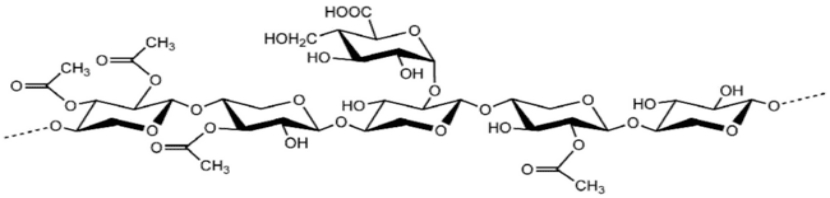  (a)Structure of hemicellulose |
| --- |
| 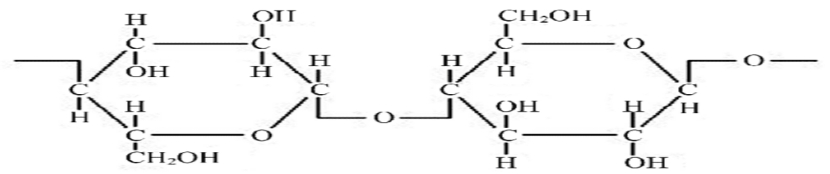  (b) Structure of cellulose |
| 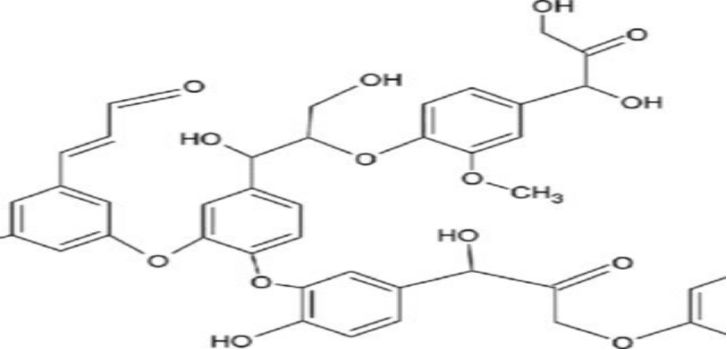    (c)Structure of lignin |

**Fig.1S:** Structure of **(a)** hemicellulose, (b) cellulose, and **(c)** lignin of corn husk**.**

**
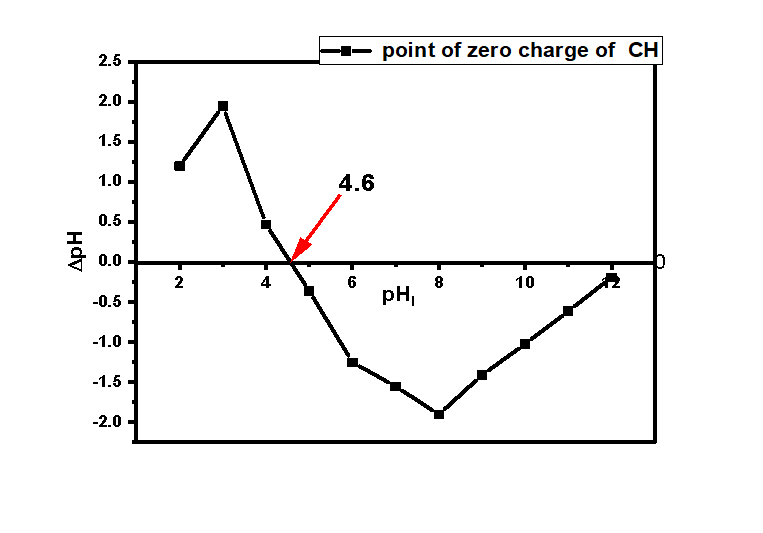
**

**Fig.2S:** Point of zero charge for CH.

| 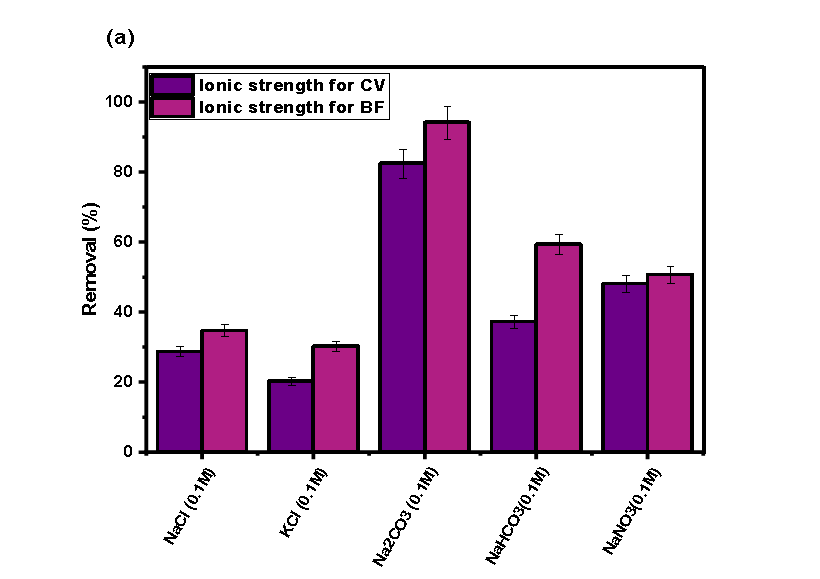 |
| --- |
| **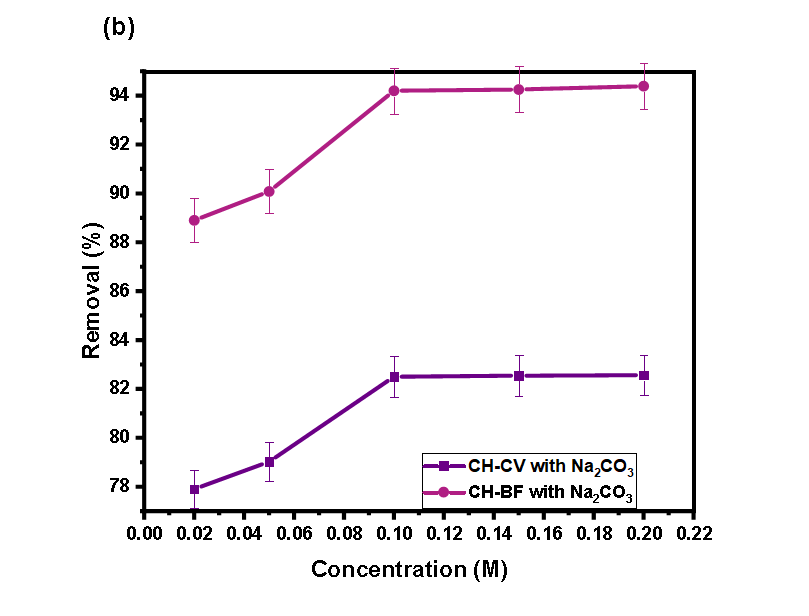** |

**Fig.3S: (a)** Effect of ionic strength on CV, and BF, and **(b)** the influence of Na_2_CO_3_ on CH-CV and CH- BF at different concentrations.
